# COVID-19 Infection During Pregnancy Induces Differential Gene Expression in Human Cord Blood Cells From Term Neonates

**DOI:** 10.3389/fped.2022.834771

**Published:** 2022-04-25

**Authors:** Suhita Gayen nee' Betal, Pedro Urday, Huda B. Al-Kouatly, Kolawole Solarin, Joanna S. Y. Chan, Sankar Addya, Rupsa C. Boelig, Zubair H. Aghai

**Affiliations:** ^1^Neonatology, Thomas Jefferson University/Nemours, Philadelphia, PA, United States; ^2^Maternal Fetal Medicine, Thomas Jefferson University, Philadelphia, PA, United States; ^3^Department of Pathology, Thomas Jefferson University, Philadelphia, PA, United States; ^4^Laboratory of Cancer Genomics, Thomas Jefferson University, Philadelphia, PA, United States

**Keywords:** global gene expression, perinatal COVID-19 exposure, Transcriptome, umbilical cord blood, infants

## Abstract

**Background:**

The COVID-19 pandemic continues worldwide with fluctuating case numbers in the United States. This pandemic has affected every segment of the population with more recent hospitalizations in the pediatric population. Vertical transmission of COVID-19 is uncommon, but reports show that there are thrombotic, vascular, and inflammatory changes in the placenta to which neonates are prenatally exposed. Individuals exposed in utero to influenza during the 1918 pandemic had increased risk for heart disease, kidney disease, diabetes, stomach disease and hypertension. Early exposure of COVID-19 during fetal life may lead to altered gene expression with potential long-term consequences.

**Objective:**

To determine if gene expression is altered in cord blood cells from term neonates who were exposed to COVID-19 during pregnancy and to identify potential gene pathways impacted by maternal COVID-19.

**Methods:**

Cord blood was collected from 16 term neonates (8 exposed to COVID-19 during pregnancy and 8 controls without exposure to COVID-19). Genome-wide gene expression screening was performed using Human Clariom S gene chips on total RNA extracted from cord blood cells.

**Results:**

We identified 510 differentially expressed genes (374 genes up-regulated, 136 genes down-regulated, fold change ≥1.5, *p*-value ≤ 0.05) in cord blood cells associated with exposure to COVID-19 during pregnancy. Ingenuity Pathway Analysis identified important canonical pathways associated with diseases such as cardiovascular disease, hematological disease, embryonic cancer and cellular development. Tox functions related to cardiotoxicity, hepatotoxicity and nephrotoxicity were also altered after exposure to COVID-19 during pregnancy.

**Conclusions:**

Exposure to COVID-19 during pregnancy induces differential gene expression in cord blood cells. The differentially expressed genes may potentially contribute to cardiac, hepatic, renal and immunological disorders in offspring exposed to COVID-19 during pregnancy. These findings lead to a further understanding of the effects of COVID-19 exposure at an early stage of life and its potential long-term consequences as well as therapeutic targets.

## Introduction

The recent coronavirus disease (COVID-19) is caused by severe acute respiratory syndrome coronavirus 2 (SARS-CoV-2). The COVID-19 pandemic has affected every segment of the population with more recent hospitalizations in the pediatric and infant population (1). The development of multisystem inflammatory syndrome in children (MIS-C) has now been well documented as a complication of SARS-CoV-2 infection. It involves a systemic inflammatory response that is thought to be due to post-infectious immune dysregulation causing macrophage stimulation and cytokine release (2, 3). Early case reports indicated that vertical transmission of COVID-19 is uncommon but have been shown to occur (4–6). However, even without vertical transmission, there is an effect on the placenta as reports of PCR-positive placentas for SARS-CoV-2 have been published (7). Additionally, infected maternal patients have shown thrombotic, vascular, and inflammatory placental changes to which neonates are prenatally exposed (7–9). A more recent study using cord blood cells reported that maternal COVID-19 infection during pregnancy affected the neonatal immune system with an increased percentage of natural killer and regulatory T cells with enhanced cytokine functionality (10). Maternal systemic inflammatory response due to COVID-19 during pregnancy as well as inflammatory changes in the placenta can incite a fetal inflammatory response, immune dysregulation, epigenetic changes and differential gene expression that could have long-term consequences in offspring. In life-course studies of individuals exposed in utero to influenza during the 1918 pandemic, there was a significant increased risk for heart disease, kidney disease, diabetes, stomach disease and hypertension when compared with the general population (11, 12). Experts have raised concerns that offspring born to mothers with COVID 19 infection during pregnancy may have similar long-term consequences (13).

Gene expression studies of cells and tissues have become a major tool for discovery in the pathogenesis of various diseases. Global gene expression by transcriptomic analysis can uncover gene signatures and help delineate the molecular pathways that could be involved in the long-term consequences to offspring born to mothers with COVID-19 infection during pregnancy. Previous study has reported differential gene expression patterns associated with exposure to histological chorioamnionitis in preterm infants (14, 15). More recently, we described differential gene expression in cord blood mononuclear leukocytes after exposure to histological chorioamnionitis in term neonates (16). Similar to histological chorioamnionitis, maternal COVID-19 infection during pregnancy can potentially incite fetal inflammatory response and differential gene expression (17).

The fetal environment has been shown to impact neonatal gene expression with long-term consequences into childhood and even adult life (18–20). One mode for this environmental impact has been attributed to epigenetic modifications that lead to differential gene expression and can increase the risk for various immunological, allergic, and chronic diseases later in life (18, 19, 21). We have previously demonstrated fetal epigenetic changes in relation to maternal chorioamnionitis and given the known inflammatory effects of COVID-19 on maternal physiology (22), the fetal environment may also be altered with downstream effects on gene expression. There is no data examining the impact of COVID-19 during pregnancy on global gene expression in neonates. Our study aims to examine the effect of COVID-19 infection during pregnancy on global gene expression modifications in neonates through cord blood cells analysis. To the best of our knowledge, this is the first study to investigate global gene expression profile in cord blood cells from neonates exposed to COVID-19 infection during pregnancy.

## Methods

### Ethical Approval

Institutional Review Board of Thomas Jefferson University Hospital had approved all human protocols and procedures described in this study. Informed consent was signed by the participants (8 COVID-19 positive mothers and 2 controls). Six control samples were used from a related study on discarded blood and placental tissue, thus the informed consent was waived for these participants. These control samples were collected from healthy infants born before the COVID-19 pandemic (August and September 2019) with no exposure to clinical or histological chorioamnionitis. All experiments performed in this study were approved by the Nemours Institutional Biosafety Committee.

### Study Design

This is a prospective observational study that examines differential gene expression in cord blood cells of term singleton infants born to mothers with or without a diagnosis of COVID-19 either during pregnancy or at delivery. Electronic medical records were reviewed for demographic, medical, and obstetric history. Mothers with clinical or histological chorioamnionitis were excluded as this may impact gene expression. Similarly, mothers who received the COVID-19 vaccine were excluded.

### Cord Blood Collection, Processing and Storage

At the time of delivery, the umbilical cord was disinfected and cut at the placental side of the clamp. 2.5 ml of cord blood was collected in PAXgene blood RNA tube (BD Catalog # 762165) and then processed and stored as per manufacturer's guidelines. In brief, collected blood was mixed gently by inverting 10 times, incubated for 2 h at room temperature, then saved overnight at −20°C and moved to −80°C next day for long term storage.

### RNA Isolation and Gene Expression Study

Total RNA was isolated using PAXgene Blood RNA Kit (PreAnalytiX, A Qiagen/BD Company, Switzerland) following manufacturer's protocol. RNA was eluted with 40 μl of elution buffer and then quantified on a Nanodrop ND-2000 spectrophotometer (Thermo Fisher Scientific, Waltham, MA), and quality was assessed by an Agilent 2200 TapeStation (Agilent Technologies, Palo Alto, CA). 100 ng of RNA was used from each sample to prepare fragmented biotin-labeled cDNA by GeneChip WT PLUS reagent kit (Affymetrix, Santa Clara, CA). Human Clariom S gene chips were hybridized with 2.5 μg of fragmented biotin-labeled cDNA in 100 μl hybridization cocktail, followed by target denaturation at 99°C for 5 min and then 45°C for 5 min. Hybridization was performed for 16 h at 45°C with a rotation of 60 rpm. GeneChip hybridization wash and stain kit was used to wash and stain the arrays in GeneChip Fluidic Station 450. Chips were scanned on GeneChip Scanner 3000 using Command Console Software (Thermo Fisher Scientific, Waltham, MA).

### Statistical Analysis

The COVID group was compared with the control group using Transcriptome Analysis Console software 4.0 (TAC 4.0, Thermo Fisher Scientific, Waltham, MA). Sst-RMA normalization was performed on 16 cel files generated from the samples. Student *t*-test was performed for comparison of the two groups. Genes with fold change ≥1.5 and *p* ≤ 0.05 were identified as differentially expressed. Gene expression data is available at the Gene Expression Omnibus (GEO) database of the NIH (Accession number GSE195938). Data were analyzed through the use of IPA (QIAGEN Inc., https://digitalinsights.qiagen.com/IPA) (23). IPA is one of advanced bioinformatic tools provided by Qiagen Inc. that is a web-based software application program for the analysis, integration, and interpretation of data derived from microarray, gene expression or other array based or sequencing methods. IPA analyses and interprets data based on the comprehensive, manually curated content of the Ingenuity Knowledge Base. IPA identifies Canonical pathways, Networks, Tox Functions and Upstream regulators.

## Results

Sixteen term infants were enrolled in this study. Eight infants had COVID-19 exposure during pregnancy (COVID-19 group), and eight infants born either before the pandemic (*n* = 6) or maternal COVID-19 antibody negative at the time of delivery (*n* = 2) served as the Control group. COVID-19 infection was diagnosed at a median of 89 days (range 1–238 days) before delivery, two mothers were symptomatic at the time of delivery, one with severe symptoms. The median gestational age of COVID-19 diagnosis was 24 weeks (range 5–37 weeks). Clinical and demographic data is depicted in Table 1.

**Table 1:** Demographic and clinical characteristics.

	**COVID-19 exposure** ***N =*** **8**	**Control** **(*N =* 8)**	***p***
Birth weight in Kg (mean ± SD)	3.14 ± 0.63	2.98 ± 0.56	0.6
Gestational age in weeks (mean ± SD)	38.1 ± 1.3	38.5 ± 1.4	0.6
Male sex *n* (%)	5 (62.5)	6 (75.0)	1.0
Maternal diabetes *n* (%)	1 (12.5)	0 (0)	1.0
Chronic hypertension *n* (%)	1 (12.5)	1 (12.5)	1.0
Preeclampsia *n* (%)	1 (12.5)	0 (0)	1.0
Small for gestational age *n* (%)	1 (12.5)	1 (12.5)	1.0
Healthy neonate *n* (%)	8 (100)	8 (100)	1.0

### Differential Gene Expression

Five hundred and ten genes (probe IDs) were found to be differentially expressed with a fold change ≥1.5 (*p* ≤ 0.05) when COVID-19 group array data was compared with the Control group using Transcriptome Analysis Console (TAC 4.0) software. Of these, 374 genes were up-regulated (Supplementary Table 1), and 136 genes were down-regulated (Supplementary Table 2). The top 10 up-regulated and down-regulated genes based on the fold change are reported in Table 2.

**Table 2:** Top 10 differentially up- or down-regulated genes associated with exposure to Covid.

**Probe ID**	**Gene symbol**	**Covid group average exp**	**Control group average exp**	**Fold CHANGE**	**Up/down**	***P*** **-value**
TC0100013223.hg.1	RAP1GAP	1,833.01	288.01	6.37	Up	0.0367
TC0400011013.hg.1	PPBP	26,801.01	4,837.35	5.52		0.0282
TC0600011232.hg.1	HIST1H1B	4,513.40	968.76	4.65		0.01
TC0200008268.hg.1	GNLY	652.58	146.02	4.47		0.0479
TC1700010447.hg.1	CCL5	5,007.93	1,323.37	3.77		0.0271
TC1500010160.hg.1	CTSH	354.59	104.69	3.38		0.0028
TC1800007360.hg.1	RAB27B	268.73	81.57	3.28		0.0176
TC0400012922.hg.1	TLR6	1,584.71	487.75	3.25		0.0354
TC0600014083.hg.1	HIST1H2AG	3,420.52	1,097.50	3.12		0.0095
TC0400011014.hg.1	CXCL5	103.25	33.59	3.06		0.0485
TC1600011312.hg.1	HBZ	4,640.29	56,266.94	−12.09	Down	0.0148
TC0X00007704.hg.1	COX7B	699.41	2,062.24	−2.94		0.0448
TC0200008351.hg.1	RPIA	4,039.61	11,585.24	−2.86		0.0484
TC1400007430.hg.1	SYNE2	39.40	105.42	−2.68		0.0222
TC0100018307.hg.1	ACKR1	14.93	37.53	−2.51		0.0411
TC0700009680.hg.1	TMEM176A	25.11	55.33	−2.19		0.0089
TC0800006692.hg.1	MSRA	39.67	85.63	−2.16		0.0061
TC0900007457.hg.1	CNTNAP3P2	59.30	127.12	−2.15		0.0279
TC1400007227.hg.1	LGALS3	4,039.61	7,858.29	−1.95		0.0194
TC0200016424.hg.1	LBH	1,052.79	2,048.00	−1.94		0.0407

### Ingenuity Pathway Analysis

**Figure 1:**
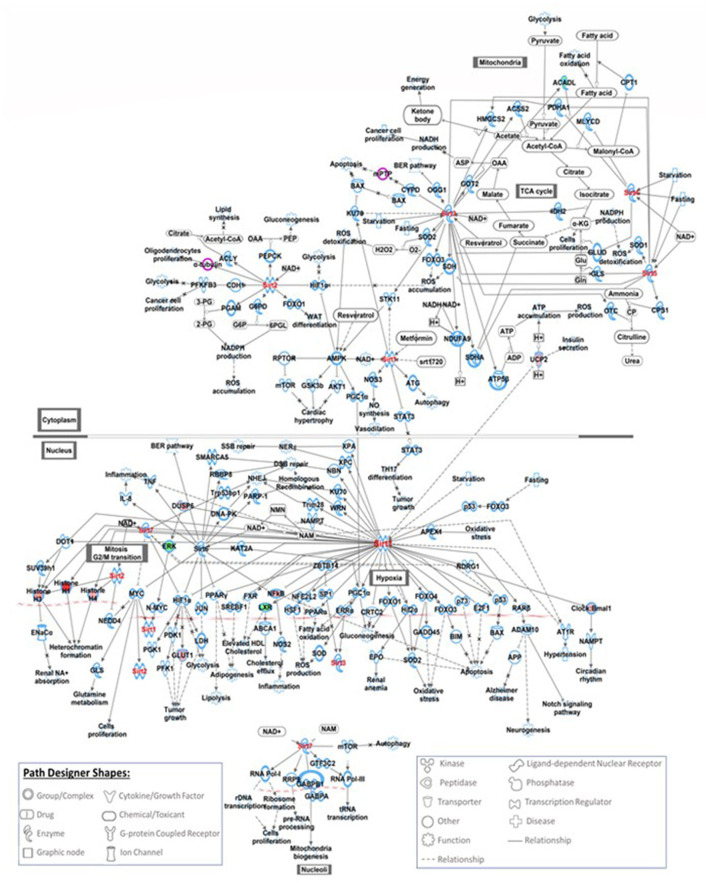
This figure shows the IPA canonical TREM1 signaling pathway. TREM1 is an important signaling receptor that plays role in systemic infections, inflammation, neurological development and coagulation. Seven genes involved in TREM-1 signaling pathways were modified with exposure to COVID-19, red filled path designer shapes are upregulated genes and green filled path designer shapes are downregulated genes.

Five hundred and ten probe sets (fold change ≥ 1.5, *p* ≤ 0.05) were used for pathway analysis using Qiagen Ingenuity Pathway Analysis (IPA) software (QIAGEN Inc., https://digitalinsights.qiagen.com/IPA) (23). Four hundred canonical pathways were identified to be modified by IPA after exposure to COVID-19 during pregnancy. Top canonical pathways altered included: sirtuin signaling (Supplementary Figure 1), DNA methylation and transcriptional repression signaling, TREM1 signaling (Figure 1), transcriptional regulatory network in embryonic stem cells and kinetochore metaphase signaling. Relevant altered canonical pathways are depicted in Table 3. Differential gene expression with COVID-19 exposure could potentially alter 53 diseases and biological functions. Top altered diseases/disorders associated with changes in gene expression after exposure to COVID-19 during pregnancy include: cancer, organismal injury and abnormalities, cardiovascular disease, connective tissue disorders, and hematological disease. Cellular and molecular functions potentially impacted by differential gene expression observed in neonates of mothers with COVID-19 included cellular assembly and organization, DNA replication, recombination, and repair, cellular movement, cellular development, cellular growth and proliferation. Physiological system development and function that may be altered by differential gene expression are hematological system development and function, immune cell trafficking, embryonic development, hematopoiesis and lymphoid tissue structure and development. Important functions that may be modified with exposure to COVID-19 are listed in Table 4.

**Table 3:** Important canonical pathways picked-up by ingenuity pathway analysis of the differentially expressed genes between Covid and control group.

**Ingenuity canonical pathways**	**-log (*p*-value)**	**Number of genes involved**	**Molecules involved in pathways**
Sirtuin signaling pathway	4.28	18	ACADL, CLOCK, DUSP6, H1-5, H3C3, H4C11, MAPK15, NDUFA1, NDUFB3, NR1H2, REL, SLC2A1, TOMM70, TUBA1B, TUBA1C, UCP2, VDAC1, VDAC3
DNA methylation and transcriptional Repression Signaling	3.11	5	H4C11,H4C12,H4C15,H4C8,H4C9
TREM1 Signaling	3.03	7	CD86,CIITA,IL1RL1,REL,TLR1,TLR6, TYROBP
Transcriptional regulatory network in embryonic stem cells	2.21	5	H4C11,H4C12,H4C15,H4C8,H4C9
Kinetochore metaphase signaling pathway	2.15	7	ARPP19,ENSA,H2AC18/H2AC19,KIF2C, MAD2L1, PLK1,ZW10
BAG2 signaling pathway	2.08	6	ANXA2,HSP90AA1,HSPA1A/HSPA1B, PSMD6,PSMD8,REL
Gα12/13 signaling	2.07	8	BTK, CDH12,GNA13,MEF2C,MEF2D,PTK2, RASA1,REL
Toll-like receptor signaling	1.59	5	IL1RL1,REL,TICAM2,TLR1,TLR6
B cell development	1.37	3	CD86, HLA-DPA1,HLA-DPB1
Antigen presentation pathway	1.31	3	CIITA, HLA-DPA1,HLA-DPB1
Remodeling of epithelial adherens junctions	1.26	4	ARPC2, DNM3,TUBA1B,TUBA1C
Natural killer cell signaling	1.22	8	CFL2,HSPA1A/HSPA1B,KLRB1,KLRC2, KLRD1,REL,STAT4,TYROBP
Neuroinflammation signaling pathway	1.16	11	CCL5, CD86,CX3CR1,HLA-DPA1, HLA-DPB1, MAPK15, REL, TICAM2, TLR1,TLR6,TYROBP
Th1 pathway	0.955	5	CD86, HLA-DPA1,HLA-DPB1, KLRD1, STAT4
Th1 and Th2 activation pathway	0.807	6	CD86,HLA-DPA1,HLA-DPB1, IL1RL1, KLRD1,STAT4
Granulocyte adhesion and diapedesis	0.752	6	CCL5, CXCL5,GNAI3,IL1RL1,PPBP,RDX
Hypoxia signaling in the cardiovascular system	0.688	3	HSP90AA1, P4HB,UBE2T
Role of pattern recognition receptors in recognition of bacteria and viruses	0.676	5	CCL5, REL,TLR1,TLR6,TNFSF14
Phagosome maturation	0.393	4	CTSH, NSF,TUBA1B,TUBA1C

**Table 4:** Modified diseases and functions obtained from ingenuity pathway analysis for the differentially expressed genes between Covid and control group.

**Modified diseases and functions**	**Range of *p*-values for genes involved**	**Number of genes involved**
Cancer	3.31E-07-6.63E-03	438
Organismal injury and abnormalities	3.31E-07-6.63E-03	438
Cellular assembly and organization	6.39E-07-6.47E-03	32
DNA replication, recombination, and repair	6.39E-07-6.47E-03	28
Cardiovascular disease	3.98E-05-5.42E-03	20
Connective tissue disorders	3.98E-05-1.39E-03	7
Hematological disease	3.98E-05-6.63E-03	125
Immunological disease	3.98E-05-6.63E-03	77
Cellular movement	6.75E-05-6.25E-03	26
Hematological system development and function	6.75E-05-5.94E-03	50
Immune cell trafficking	6.75E-05-5.94E-03	29
Inflammatory response	6.75E-05-5.94E-03	74
Cellular development	1.41E-04-4.89E-03	12
Cellular growth and proliferation	1.41E-04-6.12E-04	11
Embryonic development	1.41E-04-1.41E-04	10
Hematopoiesis	1.41E-04-6.12E-04	11
Lymphoid tissue structure and development	1.41E-04-1.92E-03	13
Cellular compromise	1.51E-04-2.37E-04	29
Cell death and survival	1.87E-04-2.07E-03	92
Cellular function and maintenance	1.93E-04-6.63E-03	41
Cell-to-cell signaling and interaction	3.02E-04-4.44E-03	33
Cell cycle	5.07E-04-6.25E-03	29
Inflammatory disease	5.22E-04-4.69E-03	8
Protein synthesis	7.05E-04-4.39E-03	45
Metabolic disease	2.32E-03-5.96E-03	15

### IPA Networks

**Figure 2:**
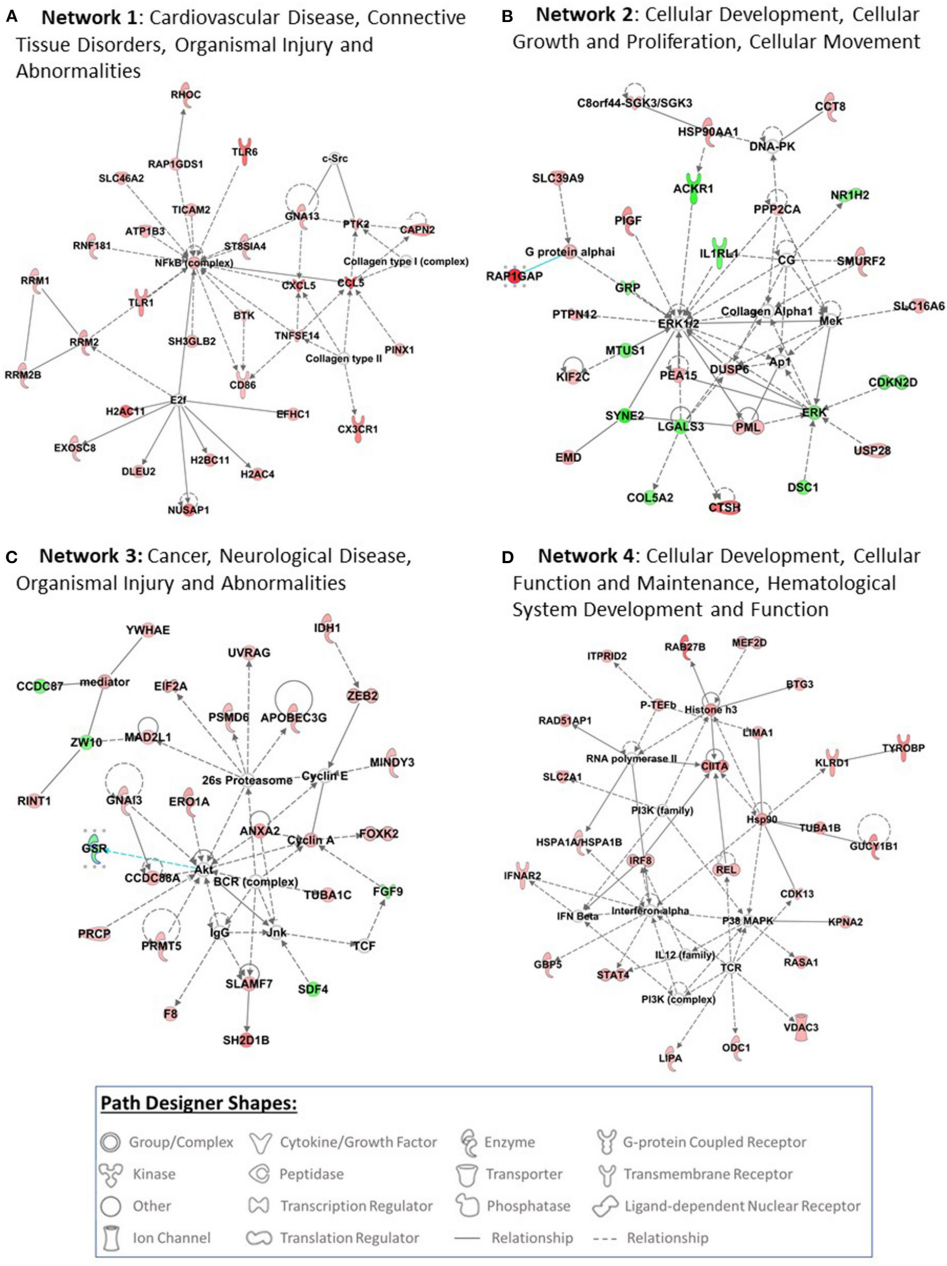
Top 4 networks that are picked up by pathway analysis are presented in this figure **(A–D)**. Pathway analysis was performed using Ingenuity Pathway Analysis (IPA) software (QIAGEN Inc., https://digitalinsights.qiagen.com/IPA) by loading the 510 probe sets that were differentially expressed with exposure to COVID-19. The red filled path designer shapes are upregulated genes and green filled path designer shapes are downregulated genes after exposure to COVID-19.

The top 4 networks that were picked up by the IPA are: Network 1—cardiovascular disease, connective tissue disorders, organismal injury and abnormalities (score 44, focus molecules 30), Network 2—cellular development, cellular growth and proliferation, cellular movement (score 37, focus molecules 27), Network 3—cancer, neurological disease, organismal injury and abnormalities (score 35, focus molecules 26), Networks 4—cellular development, cellular function and maintenance, hematological system development and function (score 31, focus molecules 24) (Figure 2).

### Tox Functions

Twenty-nine tox functions are potentially altered with COVID-19 exposure during pregnancy. Top tox functions related to cardiotoxicity include cardiac arrythmia, cardiac dilation, cardiac enlargement, congenital heart anomaly and congestive cardiac failure. Top potentially altered hepatotoxic functions are liver hyperplasia/ hyperproliferation, hepatocellular carcinoma, liver failure, and liver fibrosis. Top potentially altered tox functions related to nephrotoxicity are glomerular injury, renal fibrosis, nephrosis, kidney failure and renal damage. The potentially altered tox functions are listed in Table 5.

**Table 5:** Top tox functions modified with Covid-19 exposure.

**Top tox functions**	**Category**	**Range of *p*-values for genes involved**	**Number** **of** **molecules**
Cardiac arrythmia	Cardiotoxicity	2.17E-02-1E00	6
Cardiac dilation		2.17E-02-5.36E-01	5
Cardiac enlargement		2.17E-02-5.36E-01	5
Congenital heart anomaly		2.17E-02-3.83E-01	2
Cardiac congestive cardiac failure		1.04E-01-1.04E-01	1
Liver hyperplasia/ hyperproliferation	Hepatotoxicity	9.79E-05-5.84E-01	192
Hepatocellular carcinoma		2.23E-03-5.65E-01	52
Liver failure		2.17E-02-2.48E-01	1
Liver fibrosis		2.68E-02-1E00	7
Liver proliferation		2.68E-02-2.68E-02	2
Glomerular injury	Nephrotoxicity	4.08E-02-5.46E-01	2
Renal fibrosis		4.08E-02-1.97E-01	2
Nephrosis		4.29E-02-5.9E-01	2
Kidney failure		1.97E-01-2.39E-01	4
Renal damage		2.48E-01-2.48E-01	1

### Upstream Regulators

Upstream regulator analysis by IPA identified five key regulators of which one is a phosphatase (PDCD1), three are transcriptional regulators (E2F3, NUPR1 and LARP1) and one mitotic spindle protein (CKAP2L). E2F3, CKAP2L and LARP1 are identified to be activated regulators (z-scores 2.12, 2.24 and 2.24, respectively) and PDCD1 and NUPR1 are inhibited regulators (z-scores −2.24 and −2.4, respectively). The upstream regulators and their target molecules are depicted in Table 6.

**Table 6:** Key upstream regulators.

**Upstream regulator**	**Molecule type**	**Predicted activation state**	**Activation z-score**	***p*** **-value of overlap**	**Target molecules in dataset**
E2F3	transcription regulator	Activated	2.121	0.00352	BAIAP2L1,CCNA2,H2BC11,H2BC15, MAD2L1, PLK1,RRM2,TMPO
CKAP2L	other	Activated	2.236	0.00679	KIF2C,MAD2L1,PLK1,TMPO,TUBA1B
LARP1	translation regulator	Activated	2.236	0.0417	RPL11,RPL34,RPL36A,RPS24,RPS28
PDCD1	phosphatase	Inhibited	−2.236	0.00198	CCNA2,GNLY,KLRF1,KPNA2,SH2D1B
NUPR1	transcription regulator	Inhibited	−2.4	0.00456	C3orf62,CCNA2,CXCL5,FAM72C/FAM72D, H1-5,H2AC13,H2AC14, H2AC4, H2BC14, H3C15, H3C3,KIF2C, LMAN2L, NSF, PEA15, PLK1, SLC16A6, SLC2A1, SYNE2,TMPO,UEVLD

## Discussion

COVID-19 infection during pregnancy could potentially have deleterious short- and long-term consequences in offspring. Maternal COVID-19 infection during pregnancy leads to a maternal systemic inflammatory response and inflammatory, thrombotic, and vascular changes in the placenta. This can incite a fetal inflammatory response, immune dysregulation, epigenetic changes and differential gene expression that could portray short- and long-term effects in offspring. To our knowledge, this is the first study reporting differential gene expression profile in cord blood cells in term infants exposed to COVID-19 infection during pregnancy. We identified 510 differentially expressed genes (374 genes up-regulated, 136 genes down-regulated) in cord blood cells after exposure to COVID-19 during pregnancy. IPA identified important canonical pathways associated with diseases such as cardiovascular disease, hematological disease, cancer, embryonic and cellular development. Tox functions related to cardiotoxicity, hepatotoxicity and nephrotoxicity were also altered after exposure to COVID-19 during pregnancy. Our findings add to literature on further understanding the effects of COVID-19 exposure at an early stage of life and its potential short- and long-term consequences.

The top up-regulated genes after exposure to COVID-19 during pregnancy included ras-associated protein-1 GTPase-activating-protein (RAP1GAP), pro-platelet basic protein (PPBP), and histone cluster 1, H1b (HIST1H1B). RAP1GAP plays a key role in the control of adherens junctions at different levels that takes part in cell adhesion and cell-cell junction formation (24) and neuronal differentiation (25). In animal models, RAP1GAP mediates angiotensin II-induced cardiomyocyte hypertrophy by inhibiting autophagy and increasing oxidative stress (26). PPBP is a potent chemoattractant and activator of neutrophils. It also stimulates DNA synthesis, mitosis and glycolysis (https://www.genecards.org/cgi-bin/carddisp.pl?gene = PPBP) (27). HIST1H1B takes part in regulating individual gene transcription through chromatin remodeling, nucleosome spacing and DNA methylation (28, 29). The top down-regulated genes included; hemoglobin zeta (HBZ), cytochrome c oxidase subunit VIIb (COX7B), atypical chemokine receptor 1 (Duffy blood group) (ACKR1). HBZ is a novel hemoglobin that takes part in heme binding, iron ion binding, cellular oxidant detoxification (30) and represses viral transcription (31). COX7B plays an important role in proper central nervous system (CNS) development in vertebrates and mitochondrial electron transport (32). ACKR1 binds to several proinflammatory chemokines to control chemokine levels and regulates neutrophil counts in blood. Neutropenia in healthy individuals of African ancestry has been linked with the variant rs2814778(G) of the gene encoding ACKR1 (33). Absence of erythroid ACKR1 changes the steady-state hematopoiesis and may impact the bone marrow response during infection, inflammation, injury and cancer (33).

Understanding the effects of COVID-19 exposure on genes involved in canonical pathways could lead to better understanding of short- and long-term consequences in offspring. We have identified several key canonical pathways that are potentially modified after exposure to COVID-19 infection during pregnancy. 18 genes involved in sirtuin signaling pathways were modified with exposure to COVID-19. The sirtuin family are nicotinamide dinucleotide (NAD+)-dependent deacylases involved in metabolic regulation and are an essential factor in delaying cellular senescence and extending organismal lifespan (34). The sirtuin family is also a key regulator of acute and chronic inflammation. Multiple authors have reported the association of Sirtuin-1 (SIRT1) with COVID-19 infection and its capability to affect multi-organ failure. Miller et al. have reported that activation of SIRT1 may be a crucial factor in the prevention of the hyperinflammatory response and may be necessary for a successful defense against viral infections (35). The two other key signaling pathways modified with exposure to COVID-19 infection during pregnancy include DNA methylation and transcription repression signaling and TREM1 signaling. DNA methylation and histone modifications, two important epigenetic mechanisms, have frequently been reported to facilitate or oppose the pathogenicity of SARS-CoV-2 in human cells (36). TREM-1 is an important signaling receptor expressed on neutrophils and monocytes that plays an important role in systemic infections, inflammation, neurological development and coagulation (37). Resende et al. reported that TREM-1 and its soluble form may play a pivotal role in the pathogenesis of SARS-CoV-2 infection (38). Kerget et al. have shown that TREM-1 and TREM-2 have an important role in inflammation and may serve as biomarkers and therapeutic targets in the early treatment and follow-up of COVID-19 (39).

IPA of 510 differentially expressed genes after exposure to COVID-19 has identified several networks. Network 1 is associated with cardiovascular disease, connective tissue disorders, organismal injury and abnormalities. Cardiovascular comorbidities are commonly reported in patients with COVID-19 infection. Myocardial injury is reported in >25% of critical cases and could manifest in 2 patterns: acute myocardial injury and dysfunction on presentation and myocardial injury that develops with severity of illness (40). Cardiac complications reported in children with MIS-C include: abnormal cardiac enzymes, abnormal electrocardiographs, decreased cardiac function, coronary artery dilation and aneurysms, mitral and tricuspid valve regurgitation, aortic valve insufficiency and pericardial effusion (41). Network 2 is associated with cellular development, cellular growth and proliferation, and cellular movement. Network 3 is associated with cancer, neurological disease, organismal injury and abnormalities. Exposure to COVID-19 infection differentially expressed genes involved in cellular replication, DNA damage, metabolism, and epigenetic regulation that are also implicated in cancer pathogenesis (42–44). It is too early to suggest if COVID-19 infection increases the risk for cancer. However, emerging evidence suggest COVID-19 infection may reactivate dormant cancer cells (43).

COVID-19 exposure altered 29 tox functions in cord blood cells in our study. The top tox functions are related to cardiotoxicity (cardiac arrythmia, cardiac dilation, cardiac enlargement, congenital heart anomaly and congestive cardiac failure), hepatotoxic functions (liver hyperplasia/ hyperproliferation, hepatocellular carcinoma, liver failure and liver fibrosis), nephrotoxicity (glomerular injury, renal fibrosis, nephrosis, kidney failure and renal damage). MIS-C, a life-threatening hyperinflammatory condition is a complication of COVID-19 infection in children with multi-system organ involvement (45). Our data indicates that exposure to COVID-19 during pregnancy is associated with altered toxic functions related to cardiac, hepatic and renal dysfunction, the common organ dysfunctions seen in children with MIS-C. In an animal model of COVID-19 infection, Li et al. reported that the SARS-CoV-2 virus can shut down energy production in cells of the heart, kidneys, spleen and other organs (46).

Upstream regulator analysis identified five key regulators after exposure to COVID-19 in our study, one phosphatase (PDCD1), 3 transcriptional regulators (E2F3, NUPR1 and LARP1) and one mitotic spindle protein (CKAP2L). Of these, 3 are identified to be activated regulators (E2F3, CKAP2L and LARP1) and 2 are inhibited regulators (PDCD1 and NUPR1). Gao and co-workers have reported that programmed cell death protein 1 (PDCD1) expression in T cells, B cells, myeloid dendritic cells, and macrophages were upregulated in COVID-19 patients and correlated with the severity of infection (47). NUPR1 is a stress protein and plays a critical role in regulating the antioxidant system (48). Inactivation of NUPR1 impairs mitochondrial function and energy metabolism, increases ROS levels, and triggers a variety of cell death pathways, including apoptosis, autophagy, and necroptosis (48). NUPRI is an inhibited regulator after exposure to COVID-19 infection and may trigger cell death pathways in the fetus with potential long-term consequences. La-related protein 1 (LARP1), which is a strongly enriched viral RNA binder, restricts SARS-CoV-2 replication in infected cells and provide a global map of their direct RNA contact sites (49).

Our study has several strengths. To our knowledge, this is the first study reporting differential gene expression in cord blood cells from neonates exposed to COVID-19 infection during pregnancy. We have reported earlier and acknowledged that histological chorioamnionitis can alter gene expression in cord blood monocytes. We excluded infants with histological chorioamnionitis from this study. The classes of gene function identified play a role in known adult COVID-19 related pathology providing support that these findings may be implicated in downstream pediatric disease from maternal COVID-19 exposure. Our study also has several limitations. Our sample size of 16 neonates is small, but similar sample size have been used commonly in studies investigating differential gene expression using microarray (50, 51). There is an inherent chance of finding differences in the gene expression due to multiple comparison; however, we used 1.5 fold change (50, 52), and most significant genes/pathways identified had a much higher fold change than our a priori established threshold. We did not analyze data based on the severity of COVID-19 infection and the length of time between infection and birth due to limited sample size. We are planning to address these questions in our ongoing study using a larger sample size.

In conclusion, COVID-19 infection during pregnancy induces differential gene expression in cord blood cells from term neonates. We identified 510 differentially expressed genes, important canonical pathways, and tox functions related to cardiotoxicity, hepatotoxicity and nephrotoxicity in cord blood cells of infants exposed to COVID-19 infection during pregnancy. Future studies can further validate differential expression of target genes in a larger cohort of neonates exposed to COVID-19 infection during pregnancy. Our data may lead to understanding the role of key genes and pathways identified on the long-term sequelae related to exposure to COVID-19 infection during pregnancy. Functional studies on the identified genes and pathways could lead to the development of potential markers for the diseases caused by *in utero* exposure to COVID-19 infection and possible interventions to prevent those complications. Finally, our results highlight the importance of exploring downstream neonatal/pediatric consequences of maternal COVID-19 exposure even in the absence of direct vertical transmission of disease.

## Data Availability Statement

The datasets presented in this study can be found in online repositories. The names of the repository/repositories and Accession Number(s) can be found below: Gene Expression Omnibus, Accession Number GSE195938.

## Ethics Statement

The studies involving human participants were reviewed and approved by Institutional Review Board of Thomas Jefferson University Hospital. The patients/participants provided their written informed consent to participate in this study.

## Author Contributions

SG and PU contributed equally to concept and design, sample collection and processing, acquisition and assembly of data, data analysis and interpretation, and manuscript writing and share first authorship. SA contributed to data analysis and interpretation. JC, HA-K, RB, and KS contributed to concept and design, sample collection and processing, and manuscript writing. ZA contributed to concept and design, data analysis and interpretation, and manuscript writing. All authors have approved the version of the submitted manuscript.

## Funding

This study funded in part through Pilot Grant (ZA) through an Institutional Development Award (IDeA) from the National Institute of General Medical Sciences of the National Institutes of Health under Grant Number U54-GM104941 (PI: Hicks) and an NICHD Grant 3R21HD101127-01S1 (PI RCB). RB is supported by PhRMA Faculty Development Award.

## Conflict of Interest

The authors declare that the research was conducted in the absence of any commercial or financial relationships that could be construed as a potential conflict of interest.

## Publisher's Note

All claims expressed in this article are solely those of the authors and do not necessarily represent those of their affiliated organizations, or those of the publisher, the editors and the reviewers. Any product that may be evaluated in this article, or claim that may be made by its manufacturer, is not guaranteed or endorsed by the publisher.

## Supplementary Material

Supplementary Figure 1 Sirtuin signaling pathway presented in this figure is the top-most canonical pathway picked up by Ingenuity Pathway Analysis (IPA) software (QIAGEN Inc., https://digitalinsights.qiagen.com/IPA) by loading the 510 probe sets that were differentially expressed with exposure to COVID-19. In mammals there are 7 sirtuin members (SIRT1-7) that are shown in this pathway. 18 genes involved in sirtuin signaling pathways were modified with exposure to COVID-19, red filled path designer shapes are upregulated genes and green filled path designer shapes are downregulated genes.

Supplementary Table 1 Differentially Upregulated ProbeID or Genes in COVID Group.

Supplementary Table 2 Differentially Downregulated ProbeID or Genes in COVID Group.
