# Supplementary figures and images for: COVID-19 Infection During Pregnancy Induces Differential Gene Expression in Human Cord Blood Cells From Term Neonates
Source: Front Pediatr. 2022 Apr 25;10:834771. doi: 10.3389/fped.2022.834771 (PMC9084610; doi:10.3389/fped.2022.834771)

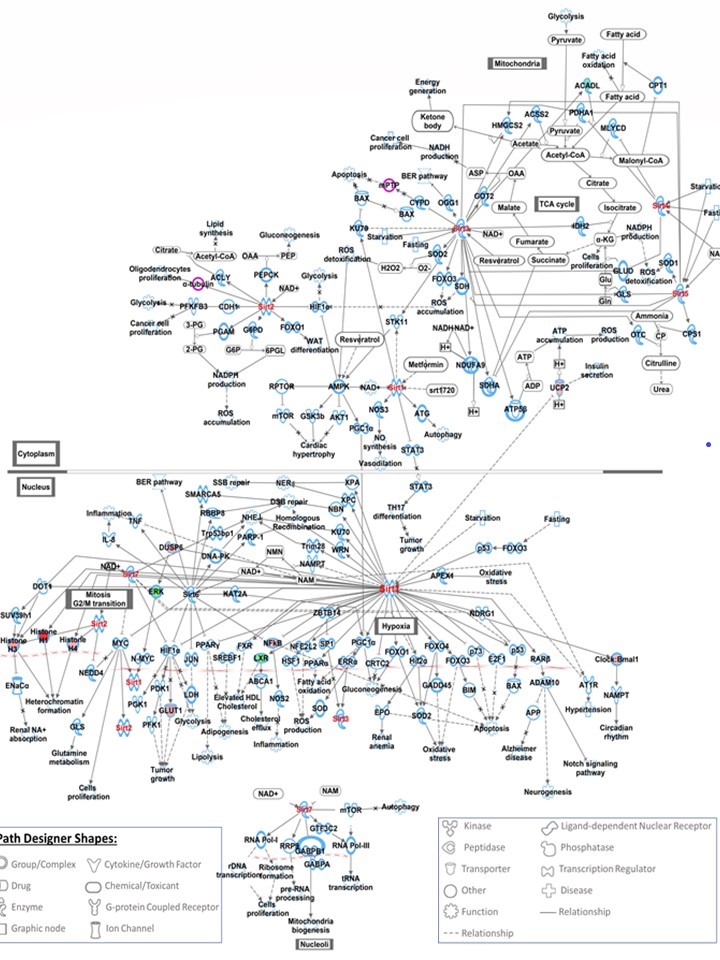

Supplement: Supplementary Figure 1 — Sirtuin signaling pathway presented in this figure is the top-most canonical pathway picked up by Ingenuity Pathway Analysis (IPA) software (QIAGEN Inc., https://digitalinsights.qiagen.com/IPA) by loading the 510 probe sets that were differentially expressed with exposure to COVID-19. In mammals there are 7 sirtuin members (SIRT1-7) that are shown in this pathway. 18 genes involved in sirtuin signaling pathways were modified with exposure to COVID-19, red filled path designer shapes are upregulated genes and green filled path designer shapes are downregulated genes. [file Image_1.JPEG]
