# Supplementary material for: COVID-19 Infection During Pregnancy Induces Differential Gene Expression in Human Cord Blood Cells From Term Neonates
Source: Front Pediatr. 2022 Apr 25;10:834771. doi: 10.3389/fped.2022.834771 (PMC9084610; doi:10.3389/fped.2022.834771)
Supplement: Supplementary Table 1 — Differentially Upregulated ProbeID or Genes in COVID Group. [file Table_1.DOCX]

**Supplementary Table-1: Differentially Upregulated ProbeID or Genes in COVID Group:**

| **Probe ID** | **Gene Symbol** | **Covid Group Average expression** | **Control Group Average expression** | **Fold Change** | **P-value** |
| --- | --- | --- | --- | --- | --- |
| TC0100013223.hg.1 | **RAP1GAP** | 1833.01 | 288.01 | **6.37** | 0.0367 |
| TC0400011013.hg.1 | **PPBP** | 26801.01 | 4837.35 | **5.52** | 0.0282 |
| TC0600011232.hg.1 | **HIST1H1B** | 4513.40 | 968.76 | **4.65** | 0.01 |
| TC0200008268.hg.1 | **GNLY** | 652.58 | 146.02 | **4.47** | 0.0479 |
| TC1700010447.hg.1 | **CCL5** | 5007.93 | 1323.37 | **3.77** | 0.0271 |
| TC1500010160.hg.1 | **CTSH** | 354.59 | 104.69 | **3.38** | 0.0028 |
| TC0400011637.hg.1 | **RPS26** | 9674.69 | 2916.45 | **3.33** | 0.0448 |
| TC1200007823.hg.1 | **RPS26** | 10586.95 | 3191.46 | **3.3** | 0.0438 |
| TC1800007360.hg.1 | **RAB27B** | 268.73 | 81.57 | **3.28** | 0.0176 |
| TC0400012922.hg.1 | **TLR6** | 1584.71 | 487.75 | **3.25** | 0.0354 |
| TC0600014083.hg.1 | **HIST1H2AG** | 3420.52 | 1097.50 | **3.12** | 0.0095 |
| TC0400011014.hg.1 | **CXCL5** | 103.25 | 33.59 | **3.06** | 0.0485 |
| TC1100010962.hg.1 | **MPEG1** | 2304.12 | 765.36 | **3.01** | 0.0312 |
| TC1500006999.hg.1 | **NUSAP1** | 192.67 | 65.34 | **2.94** | 0.0016 |
| TC0600007377.hg.1 | **HIST1H2BM** | 404.50 | 138.14 | **2.94** | 0.0193 |
| TC0300012686.hg.1 | **SLC9A9** | 266.87 | 93.70 | **2.83** | 0.0119 |
| TC1200008224.hg.1 | **GLIPR1** | 8964.45 | 3191.46 | **2.82** | 0.0091 |
| TC1400007161.hg.1 | **PTGDR** | 72.00 | 26.35 | **2.74** | 0.0102 |
| TC1200007418.hg.1 | **ANO6** | 820.30 | 300.25 | **2.73** | 0.0101 |
| TC0300010775.hg.1 | **CX3CR1** | 1978.24 | 729.11 | **2.7** | 0.0051 |
| TC0100016206.hg.1 | **SH2D1B** | 259.57 | 96.34 | **2.69** | 0.024 |
| TC1900010503.hg.1 | **TYROBP** | 21321.18 | 8135.41 | **2.61** | 0.0438 |
| TC0600011517.hg.1 | **HLA-DPA1** | 3492.39 | 1332.57 | **2.61** | 0.0058 |
| TC0500008479.hg.1 | **SNX2** | 4938.99 | 1910.85 | **2.58** | 0.0297 |
| TC0500011752.hg.1 | **FEM1C** | 61.39 | 24.08 | **2.55** | 0.0026 |
| TC0100010609.hg.1 | **DNM3** | 44.63 | 17.51 | **2.55** | 0.012 |
| TC1700008104.hg.1 | **NSF** | 280.14 | 109.90 | **2.54** | 0.0374 |
| TC1100008518.hg.1 | **ACER3** | 125.37 | 49.87 | **2.51** | 0.0008 |
| TC1400006686.hg.1 | **CMTM5** | 71.51 | 28.64 | **2.51** | 0.0082 |
| TC0600011142.hg.1 | **HIST1H3G** | 249.00 | 99.04 | **2.51** | 0.0365 |
| TC0800010685.hg.1 | **MYBL1** | 30.91 | 12.30 | **2.51** | 0.0103 |
| TC0400009088.hg.1 | **GUCY1B3** | 112.21 | 45.25 | **2.48** | 0.0031 |
| TC0200012489.hg.1 | **PIGF** | 73.52 | 30.27 | **2.43** | 0.0119 |
| TC1000008406.hg.1 | **KIF20B** | 106.89 | 44.63 | **2.39** | 0.0029 |
| TC1900007085.hg.1 | **ZNF791** | 179.77 | 75.06 | **2.39** | 0.0129 |

| **Probe ID** | **Gene Symbol** | **Covid Group Average expression** | **Control Group Average expression** | **Fold Change** | **P-value** |
| --- | --- | --- | --- | --- | --- |
| TC1600006888.hg.1 | **CIITA** | 232.32 | 99.04 | **2.35** | 0.0358 |
| TC0100015509.hg.1 | **FAM72B** | 68.12 | 28.84 | **2.35** | 0.009 |
| TC1500009109.hg.1 | **C15orf57; MRPL42P5** | 484.38 | 205.07 | **2.35** | 0.0408 |
| TC0600007282.hg.1 | **HIST1H2BF** | 178.53 | 76.11 | **2.34** | 0.0028 |
| TC0400012921.hg.1 | **TLR1** | 1530.73 | 652.58 | **2.34** | 0.0225 |
| TC0600011225.hg.1 | **HIST1H2AJ** | 63743.91 | 27364.16 | **2.33** | 0.0087 |
| TC0100016952.hg.1 | **UBE2T** | 25.81 | 11.08 | **2.33** | 0.0029 |
| TC0100011721.hg.1 | **CAPN2** | 855.13 | 372.22 | **2.3** | 0.001 |
| TC0500013191.hg.1 | **NSA2** | 962.07 | 421.68 | **2.27** | 0.0159 |
| TC0600011227.hg.1 | **HIST1H4K** | 765.36 | 340.14 | **2.26** | 0.0041 |
| TC1100013014.hg.1 | **MS4A7** | 442.64 | 195.36 | **2.26** | 0.0097 |
| TC0900009685.hg.1 | **HACD4** | 5996.90 | 2683.69 | **2.23** | 0.0264 |
| TC0100010887.hg.1 | **C1orf21** | 98.36 | 44.32 | **2.22** | 0.032 |
| TC1700012073.hg.1 | **P4HB** | 464.65 | 213.78 | **2.18** | 0.0313 |
| TC0600007265.hg.1 | **HIST1H3C** | 18.51 | 8.51 | **2.18** | 0.0202 |
| TC0600007290.hg.1 | **HIST1H2BH** | 442.64 | 205.07 | **2.16** | 0.007 |
| TC0100010454.hg.1 | **MGST3** | 1160.07 | 541.19 | **2.15** | 0.0409 |
| TC0500011751.hg.1 | **FEM1C** | 352.14 | 165.42 | **2.13** | 0.0423 |
| TC0700011584.hg.1 | **FGL2** | 5256.91 | 2469.49 | **2.13** | 0.0131 |
| TC1200012753.hg.1 | **KLRC2** | 288.01 | 137.19 | **2.11** | 0.0123 |
| TC1400008919.hg.1 | **CFL2** | 36.76 | 17.51 | **2.11** | 0.0032 |
| TC1100013022.hg.1 | **FADS2** | 1234.75 | 588.13 | **2.11** | 0.0089 |
| TC0600011125.hg.1 | **HIST1H2AB** | 744.43 | 352.14 | **2.11** | 0.0184 |
| TC0400008228.hg.1 | **METAP1** | 165.42 | 78.25 | **2.11** | 0.0006 |
| TC0500009690.hg.1 | **RUFY1** | 272.48 | 130.69 | **2.09** | 0.0044 |
| TC0100018463.hg.1 | **RHOC** | 57.68 | 27.47 | **2.09** | 0.0027 |
| TC0400007580.hg.1 | **CEP135** | 101.83 | 49.18 | **2.07** | 0.0137 |
| TC1500009636.hg.1 | **ANXA2** | 2740.08 | 1332.57 | **2.06** | 0.0138 |
| TC0900012218.hg.1 | **KLHL9** | 71.01 | 34.54 | **2.05** | 0.0003 |
| TC1100008886.hg.1 | **TMEM133** | 25.63 | 12.47 | **2.05** | 0.0038 |
| TC1400010257.hg.1 | **HSP90AA1** | 4837.35 | 2385.37 | **2.04** | 0.0046 |
| TC1200010616.hg.1 | **TUBA1B** | 15716.58 | 7696.57 | **2.04** | 0.0153 |
| TC1400009186.hg.1 | **ERO1A** | 4269.94 | 2091.03 | **2.03** | 0.0468 |
| TC0100009870.hg.1 | **HIST2H4B; HIST2H4A** | 891.44 | 436.55 | **2.03** | 0.0056 |
| TC1200006788.hg.1 | **KLRD1** | 739.29 | 367.09 | **2.02** | 0.0438 |
| TC1600007235.hg.1 | **PLK1** | 207.94 | 103.25 | **2.02** | 0.0337 |
| TC0200010788.hg.1 | **ARPC2** | 12854.63 | 6382.92 | **2.01** | 0.0148 |

| **Probe ID** | **Gene Symbol** | **Covid Group Average expression** | **Control Group Average expression** | **Fold Change** | **P-value** |
| --- | --- | --- | --- | --- | --- |
| TC1200006773.hg.1 | **KLRF1** | 134.36 | 66.72 | **2.01** | 0.0354 |
| TC1400007890.hg.1 | **GPR65** | 2352.53 | 1168.14 | **2.01** | 0.0328 |
| TC1500007056.hg.1 | **ADAL** | 48.50 | 24.08 | **2.01** | 0.008 |
| TC0100017094.hg.1 | **FAM72A** | 70.52 | 35.02 | **2.01** | 0.0299 |
| TC0600007380.hg.1 | **HIST1H2AL; HIST1H2BN** | 148.06 | 74.03 | **2** | 0.021 |
| TC1200007251.hg.1 | **KIAA1551** | 4513.40 | 2256.70 | **2** | 0.0315 |
| TC1400007554.hg.1 | **SLC39A9** | 76.11 | 37.79 | **2** | 0.0162 |
| TC0200008071.hg.1 | **MTHFD2** | 203.66 | 102.54 | **1.99** | 0.0051 |
| TC1200007430.hg.1 | **SCAF11** | 600.49 | 302.33 | **1.98** | 0.011 |
| TC0600011185.hg.1 | **HIST1H2BK** | 2368.90 | 1200.98 | **1.97** | 0.0411 |
| TC0300011842.hg.1 | **TOMM70A** | 73.52 | 37.27 | **1.97** | 0.0212 |
| TC0700013428.hg.1 | **PILRB; STAG3L5P; PVRIG2P; MIR6840; STAG3L5P-PVRIG2P-PILRB** | 584.07 | 296.11 | **1.97** | 0.0062 |
| TC0700013538.hg.1 | **TRGJ1; TRGC2; TRGJ2; TRGV9; TRGC1; TRGJP; TRGJP2** | 885.29 | 448.82 | **1.97** | 0.0343 |
| TC0X00008828.hg.1 | **EMD** | 207.94 | 106.15 | **1.96** | 0.0104 |
| TC0300006993.hg.1 | **CRTAP** | 556.41 | 284.05 | **1.96** | 0.0306 |
| TC0600011184.hg.1 | **HIST1H2BJ** | 1038.29 | 530.06 | **1.96** | 0.0196 |
| TC1300008137.hg.1 | **TMCO3** | 106.89 | 54.95 | **1.95** | 0.0112 |
| TC0500008014.hg.1 | **RASA1** | 335.46 | 172.45 | **1.95** | 0.0098 |
| TC0300010035.hg.1 | **SENP5** | 64.45 | 33.13 | **1.95** | 0.0119 |
| TC0400008985.hg.1 | **RPS3A** | 21.71 | 11.08 | **1.95** | 0.01 |
| TC0600008156.hg.1 | **ENPP4** | 162.02 | 83.29 | **1.94** | 0.0139 |
| TC0500013232.hg.1 | **SMAD5** | 401.71 | 206.50 | **1.94** | 0.0037 |
| TC1200011711.hg.1 | **GNPTAB** | 330.84 | 172.45 | **1.93** | 0.0159 |
| TC0700013609.hg.1 | **TFEC** | 108.38 | 56.10 | **1.93** | 0.0197 |
| TC0200006677.hg.1 | **RRM2** | 121.94 | 63.12 | **1.93** | 0.0405 |
| TC0200008281.hg.1 | **PTCD3** | 333.14 | 172.45 | **1.93** | 0.0042 |
| TC0100009877.hg.1 | **HIST2H3A** | 162.02 | 85.04 | **1.92** | 0.0428 |
| TC0100015701.hg.1 | **HIST2H3A; HIST2H3C** | 157.59 | 82.71 | **1.91** | 0.0143 |
| TC0600011228.hg.1 | **HIST1H2AK** | 54.19 | 28.64 | **1.9** | 0.0019 |
| TC1200008706.hg.1 | **KIAA1033** | 1552.09 | 814.63 | **1.9** | 0.03 |
| TC0300012346.hg.1 | **RPN1** | 19349.38 | 10226.32 | **1.9** | 0.0151 |

| **Probe ID** | **Gene Symbol** | **Covid Group Average expression** | **Control Group Average expression** | **Fold Change** | **P-value** |
| --- | --- | --- | --- | --- | --- |
| TC0600007278.hg.1 | **HIST1H2BE** | 93.70 | 49.18 | **1.9** | 0.0484 |
| TC1200011894.hg.1 | **C12orf76** | 31.34 | 16.45 | **1.9** | 0.0154 |
| TC0700013615.hg.1 | **ZNF800** | 1734.13 | 916.51 | **1.9** | 0.0388 |
| TC1700011451.hg.1 | **SMURF2** | 158.68 | 84.45 | **1.89** | 0.0342 |
| TC0600011235.hg.1 | **HIST1H2AM; HIST1H3J** | 190.02 | 100.43 | **1.89** | 0.0091 |
| TC1400010612.hg.1 | **PCNXL4** | 349.71 | 184.82 | **1.89** | 3.14E-05 |
| TC0200015578.hg.1 | **IDH1** | 127.12 | 67.65 | **1.88** | 0.0403 |
| TC0100010310.hg.1 | **SLAMF7** | 45.25 | 24.25 | **1.87** | 0.0411 |
| TC0600014261.hg.1 | **DDX39B; SNORD84** | 3468.27 | 1845.76 | **1.87** | 0.0478 |
| TC0600011135.hg.1 | **HIST1H3D; HIST1H2AD** | 93.70 | 50.56 | **1.86** | 0.0036 |
| TC0100013908.hg.1 | **SLC2A1** | 16844.62 | 9089.59 | **1.86** | 0.0377 |
| TC0800012001.hg.1 | **PTK2** | 51.98 | 27.86 | **1.86** | 0.0279 |
| TC1500009458.hg.1 | **ARPP19** | 261.38 | 140.07 | **1.86** | 0.0054 |
| TC2100008390.hg.1 | **PTTG1IP** | 719.08 | 388.02 | **1.85** | 0.0103 |
| TC1100012262.hg.1 | **RDX** | 115.36 | 62.25 | **1.85** | 0.0103 |
| TC1900008013.hg.1 | **PSMD8** | 596.34 | 324.03 | **1.85** | 0.0042 |
| TC0600011233.hg.1 | **HIST1H3I** | 648.07 | 349.71 | **1.85** | 0.0019 |
| TC1900008860.hg.1 | **LENG8** | 1618.00 | 873.10 | **1.85** | 0.0408 |
| TC0100015550.hg.1 | **FAM72C** | 77.17 | 42.22 | **1.84** | 0.0204 |
| TC0200010021.hg.1 | **H3F3AP4; H3F3A; H3F3B** | 69754.56 | 37902.36 | **1.84** | 0.0159 |
| TC0500010511.hg.1 | **C5orf42** | 41.36 | 22.47 | **1.84** | 0.0153 |
| TC0800010427.hg.1 | **RB1CC1** | 776.05 | 421.68 | **1.84** | 0.0199 |
| TC1500007513.hg.1 | **LACTB** | 194.01 | 106.15 | **1.83** | 0.0024 |
| TC0100010546.hg.1 | **BLZF1** | 321.80 | 176.07 | **1.83** | 0.0192 |
| TC0100014543.hg.1 | **DEPDC1** | 48.17 | 26.35 | **1.83** | 0.0424 |
| TC0100010284.hg.1 | **PEA15** | 199.47 | 109.14 | **1.83** | 0.0065 |
| TC0300007194.hg.1 | **ABHD5** | 188.71 | 103.25 | **1.83** | 0.0182 |
| TC0100015707.hg.1 | **HIST2H4A; HIST2H4B** | 781.44 | 430.54 | **1.82** | 0.0107 |
| TC0600007384.hg.1 | **HIST1H2BO** | 17.15 | 9.45 | **1.82** | 0.0068 |
| TC0300009167.hg.1 | **EIF2A** | 324.03 | 177.29 | **1.82** | 0.0064 |
| TC0700008662.hg.1 | **ARMC10** | 191.34 | 104.69 | **1.82** | 0.0011 |
| TC1700011558.hg.1 | **SLC16A6** | 130.69 | 71.51 | **1.82** | 0.0341 |
| TC0700007859.hg.1 | **TYW1** | 52.35 | 29.04 | **1.81** | 0.0496 |
| TC2100007854.hg.1 | **CCT8** | 699.41 | 385.34 | **1.81** | 0.0314 |
| TC0900006539.hg.1 | **JAK2** | 266.87 | 148.06 | **1.81** | 0.0374 |

| **Probe ID** | **Gene Symbol** | **Covid Group Average expression** | **Control Group Average expression** | **Fold Change** | **P-value** |
| --- | --- | --- | --- | --- | --- |
| TC0500012059.hg.1 | **VDAC1** | 256.00 | 142.02 | **1.8** | 0.0208 |
| TC0200007115.hg.1 | **CLIP4** | 96.34 | 53.45 | **1.8** | 0.0115 |
| TSUnmapped00000293.hg.1 | **RCC2** | 347.29 | 192.67 | **1.8** | 0.0227 |
| TC1500009429.hg.1 | **DMXL2** | 617.37 | 342.51 | **1.8** | 0.0421 |
| TC0600007285.hg.1 | **HIST1H2AE** | 4124.49 | 2288.20 | **1.8** | 0.0132 |
| TC1200006561.hg.1 | **RAD51AP1** | 12.82 | 7.21 | **1.79** | 0.0196 |
| TC1200009872.hg.1 | **KLRB1** | 218.27 | 121.94 | **1.79** | 0.0359 |
| TC0600007293.hg.1 | **HIST1H2BI** | 77.71 | 43.41 | **1.79** | 0.0052 |
| TC1600008144.hg.1 | **C16orf70** | 190.02 | 106.15 | **1.79** | 0.0164 |
| TC1500009621.hg.1 | **GTF2A2** | 120.26 | 67.18 | **1.79** | 0.0016 |
| TC1400007221.hg.1 | **SOCS4** | 142.02 | 79.34 | **1.79** | 0.0189 |
| TC1000011807.hg.1 | **SMNDC1** | 436.55 | 245.57 | **1.78** | 0.026 |
| TC1900011919.hg.1 | **ZNF708** | 445.72 | 250.73 | **1.78** | 0.0242 |
| TC0200012643.hg.1 | **CCDC88A** | 330.84 | 186.11 | **1.78** | 0.0097 |
| TC0200007015.hg.1 | **RAB10** | 5042.77 | 2817.11 | **1.78** | 0.0116 |
| TC1200011470.hg.1 | **DUSP6** | 410.15 | 229.13 | **1.78** | 0.0076 |
| TC0600007378.hg.1 | **HIST1H4J** | 6295.04 | 3565.78 | **1.77** | 0.0012 |
| TC0200013943.hg.1 | **SLC35F5** | 352.14 | 199.47 | **1.77** | 0.0415 |
| TC0300012664.hg.1 | **GK5** | 144.01 | 81.57 | **1.77** | 0.0116 |
| TC1100012358.hg.1 | **USP28** | 71.51 | 40.50 | **1.77** | 0.0176 |
| TC0900009404.hg.1 | **PUM3** | 99.73 | 56.10 | **1.77** | 0.0066 |
| TC0100014286.hg.1 | **USP24** | 19.29 | 10.93 | **1.76** | 0.011 |
| TC0100014857.hg.1 | **GBP5** | 110.66 | 62.68 | **1.76** | 0.015 |
| TC0900011669.hg.1 | **SH3GLB2** | 1287.18 | 734.19 | **1.76** | 0.0078 |
| TC0700006783.hg.1 | **TSPAN13** | 131.60 | 74.54 | **1.76** | 0.016 |
| TC1900009442.hg.1 | **TNFSF14** | 112.99 | 64.89 | **1.75** | 0.0109 |
| TC2200009274.hg.1 | **APOBEC3G** | 154.34 | 88.65 | **1.75** | 0.0258 |
| TC0600011880.hg.1 | **GTPBP2** | 401.71 | 230.72 | **1.75** | 0.0458 |
| TC0800007440.hg.1 | **VDAC3** | 929.30 | 530.06 | **1.75** | 0.0075 |
| TC0900011241.hg.1 | **SLC46A2** | 18.64 | 10.63 | **1.75** | 0.0184 |
| TC0600013147.hg.1 | **ARHGAP18** | 106.89 | 60.97 | **1.75** | 0.029 |
| TC1300008938.hg.1 | **DLEU2; MIR15A; MIR16-1; MIR3613** | 1562.89 | 903.89 | **1.74** | 0.0122 |
| TC1200008568.hg.1 | **TMPO** | 724.08 | 418.77 | **1.74** | 0.0126 |
| TC0200014631.hg.1 | **PRPF40A** | 508.46 | 292.04 | **1.74** | 0.0307 |
| TC0200013112.hg.1 | **AUP1** | 765.36 | 439.59 | **1.74** | 0.0186 |

| **Probe ID** | | **Gene Symbol** | **Covid Group Average expression** | | **Control Group Average expression** | | **Fold Change** | | **P-value** | |
| --- | --- | --- | --- | --- | --- | --- | --- | --- | --- | --- |
| TC1500007034.hg.1 | | **SNAP23** | 3821.70 | | 2194.99 | | **1.74** | | 0.016 | |
| TC0600014233.hg.1 | | **SSR1** | 2304.12 | | 1332.57 | | **1.73** | | 0.0218 | |
| TC0200015246.hg.1 | | **STAT4** | 238.86 | | 139.10 | | **1.73** | | 0.0158 | |
| TC0600008255.hg.1 | | **EFHC1** | 14.72 | | 8.51 | | **1.73** | | 0.0037 | |
| TC0400012938.hg.1 | | **RASGEF1B** | 42.81 | | 24.93 | | **1.72** | | 0.0379 | |
| TC0400012992.hg.1 | | **MFAP3L** | 34.30 | | 19.97 | | **1.72** | | 0.0009 | |
| TC0200010164.hg.1 | | **SSFA2** | 31.34 | | 18.25 | | **1.72** | | 0.018 | |
| TC0300014052.hg.1 | | **RYK** | 259.57 | | 152.22 | | **1.71** | | 0.0396 | |
| TC0600013523.hg.1 | | **PPIL4** | 107.63 | | 63.12 | | **1.71** | | 0.0308 | |
| TC0200013535.hg.1 | | **LMAN2L** | 21.71 | | 12.73 | | **1.71** | | 0.0092 | |
| TC0900007663.hg.1 | | **CEP78** | 75.06 | | 44.02 | | **1.71** | | 0.0366 | |
| TC1100011614.hg.1 | | **KCNE3** | 70.03 | | 41.07 | | **1.71** | | 0.0492 | |
| TC1600011355.hg.1 | | **NPIPA1** | 843.36 | | 491.14 | | **1.71** | | 0.0029 | |
| TC0200006524.hg.1 | | **TRAPPC12** | 317.37 | | 187.40 | | **1.7** | | 0.0012 | |
| TC1400006821.hg.1 | | **G2E3** | 65.34 | | 38.59 | | **1.7** | | 0.0291 | |
| TC2200008687.hg.1 | | **TMEM184B** | 21.41 | | 12.64 | | **1.7** | | 0.0401 | |
| TC1300008388.hg.1 | | **MTMR6** | 73.01 | | 42.81 | | **1.7** | | 0.0225 | |
| TC1300008181.hg.1 | | **CHAMP1** | 85.04 | | 49.87 | | **1.7** | | 0.0165 | |
| TC0200008244.hg.1 | | **TGOLN2** | 11.71 | | 6.92 | | **1.7** | | 0.0063 | |
| TC0700013347.hg.1 | | **AQP1** | 230.72 | | 137.19 | | **1.69** | | 0.0067 | |
| TC0600007677.hg.1 | | **HLA-DPB1** | 1226.22 | | 724.08 | | **1.69** | | 0.0171 | |
| TC1900012025.hg.1 | | **ZNF28** | 129.79 | | 76.64 | | **1.69** | | 0.01 | |
| TC1700011476.hg.1 | | **GNA13** | 3666.02 | | 2164.77 | | **1.69** | | 0.0152 | |
| TC0100015160.hg.1 | | **SLC25A24** | 123.64 | | 73.01 | | **1.69** | | 0.0208 | |
| TC0600012240.hg.1 | | **LMBRD1** | 3666.02 | | 2194.99 | | **1.68** | | 0.0247 | |
| TC0300012315.hg.1 | | **TPRA1** | 109.90 | | 65.34 | | **1.68** | | 0.0123 | |
| TC0300013977.hg.1 | | **C3orf62** | 107.63 | | 64.00 | | **1.68** | | 0.0318 | |
| TC0700008141.hg.1 | | **PTPN12** | 519.15 | | 308.69 | | **1.68** | | 0.0363 | |
| TC1600007112.hg.1 | | **CCP110** | 84.45 | | 50.21 | | **1.68** | | 0.0036 | |
| TC0300012118.hg.1 | | **TMEM39A** | 247.28 | | 148.06 | | **1.67** | | 0.013 | |
| TC1400009066.hg.1 | | **MIS18BP1** | 326.29 | | 195.36 | | **1.67** | | 0.0106 | |
| TC1000011050.hg.1 | | **AP3M1** | 617.37 | | 369.65 | | **1.67** | | 0.0249 | |
| TC1400009329.hg.1 | | **RTN1** | 41.07 | | 24.59 | | **1.67** | | 0.0283 | |
| TC0500011754.hg.1 | | **TMED7-TICAM2; TICAM2; TMED7** | 996.00 | | 596.34 | | **1.67** | | 0.0309 | |
| TC2000007620.hg.1 | | **ARFGEF2** | 222.86 | | 133.44 | | **1.67** | | 0.0112 | |
| TC0900006961.hg.1 | | **DNAJA1** | 4039.61 | | 2418.67 | | **1.67** | | 0.0065 | |
| **Probe ID** | **Gene Symbol** | | | **Covid Group Average expression** | | **Control Group Average expression** | **Fold Change** | **P-value** | |  |
| TC0200016757.hg.1 | **NCKAP1** | | | 37.79 | | 22.63 | **1.67** | 0.0157 | |  |
| TC0800012386.hg.1 | **PINX1; MIR1322** | | | 89.88 | | 54.19 | **1.66** | 0.0175 | |  |
| TC1300006914.hg.1 | **UFM1** | | | 242.19 | | 146.02 | **1.66** | 0.0496 | |  |
| TC0500012965.hg.1 | **LMAN2** | | | 1820.35 | | 1097.50 | **1.66** | 0.0263 | |  |
| TC0600007374.hg.1 | **HIST1H2AI** | | | 3468.27 | | 2076.59 | **1.66** | 0.0258 | |  |
| TC0100009723.hg.1 | **FAM72D** | | | 54.57 | | 32.90 | **1.66** | 0.0273 | |  |
| TC0500011579.hg.1 | **ST8SIA4** | | | 2574.36 | | 1552.09 | **1.66** | 0.0493 | |  |
| TC0100009035.hg.1 | **KIAA1107** | | | 15.89 | | 9.58 | **1.66** | 0.0467 | |  |
| TC2000007819.hg.1 | **CSTF1** | | | 135.30 | | 81.57 | **1.66** | 0.0431 | |  |
| TC0400012150.hg.1 | **TMEM154** | | | 9475.59 | | 5712.87 | **1.66** | 0.0472 | |  |
| TC0100015761.hg.1 | **CERS2** | | | 213.78 | | 129.79 | **1.65** | 0.0147 | |  |
| TC1200011311.hg.1 | **NAP1L1** | | | 9877.98 | | 5996.90 | **1.65** | 0.0139 | |  |
| TC1600006628.hg.1 | **KCTD5** | | | 837.53 | | 504.95 | **1.65** | 0.0264 | |  |
| TC0700012296.hg.1 | **C7orf60** | | | 138.14 | | 83.87 | **1.65** | 0.0117 | |  |
| TC0100009876.hg.1 | **HIST2H2AA3; HIST2H2AA4** | | | 1341.84 | | 820.30 | **1.64** | 0.0145 | |  |
| TC1000011005.hg.1 | **P4HA1** | | | 233.94 | | 143.01 | **1.64** | 0.0326 | |  |
| TC0300013609.hg.1 | **LSG1** | | | 272.48 | | 166.57 | **1.64** | 0.0369 | |  |
| TC0500012162.hg.1 | **BRD8** | | | 179.77 | | 109.14 | **1.64** | 0.0201 | |  |
| TC0600010757.hg.1 | **SLC35B3** | | | 81.01 | | 49.87 | **1.63** | 0.0415 | |  |
| TC0200013332.hg.1 | **PLGLB1** | | | 184.82 | | 113.77 | **1.63** | 0.0474 | |  |
| TC0500009863.hg.1 | **LPCAT1** | | | 195.36 | | 120.26 | **1.63** | 0.0337 | |  |
| TC0600012764.hg.1 | **SEC63** | | | 617.37 | | 380.04 | **1.63** | 0.0166 | |  |
| TC0100016676.hg.1 | **EDEM3** | | | 704.28 | | 433.53 | **1.63** | 0.0224 | |  |
| TC1100010495.hg.1 | **LMO2** | | | 1067.48 | | 657.11 | **1.63** | 0.0222 | |  |
| TC1300006890.hg.1 | **EXOSC8** | | | 97.68 | | 60.13 | **1.63** | 0.0283 | |  |
| TC0300009600.hg.1 | **ZNF639** | | | 143.01 | | 87.43 | **1.63** | 0.0086 | |  |
| TC1900011692.hg.1 | **ZNF429** | | | 67.18 | | 41.36 | **1.63** | 0.007 | |  |
| TC1200008877.hg.1 | **SH2B3** | | | 61.82 | | 38.05 | **1.63** | 0.0414 | |  |
| TC2100008494.hg.1 | **IFNAR2** | | | 6122.90 | | 3743.05 | **1.63** | 0.0249 | |  |
| TC0900008312.hg.1 | **SLC44A1** | | | 88.03 | | 53.82 | **1.63** | 0.0393 | |  |
| TC1100010092.hg.1 | **EIF4G2; SNORD97** | | | 29944.43 | | 18305.63 | **1.63** | 0.0152 | |  |
| TC1900011312.hg.1 | **ZNF611** | | | 1176.27 | | 719.08 | **1.63** | 0.0211 | |  |
| TC1600008164.hg.1 | **FAM65A** | | | 247.28 | | 153.28 | **1.62** | 0.0255 | |  |
| TC1900011794.hg.1 | **ZNF701; ZNF137P** | | | 522.76 | | 321.80 | **1.62** | 0.017 | |  |
| TC1100011602.hg.1 | **UCP2** | | | 161368.56 | | 100024.92 | **1.62** | 0.0157 | |  |
| TC1900010533.hg.1 | **ZNF850** | | | 55.33 | | 34.30 | **1.62** | 0.0269 | |  |

| **Probe ID** | **Gene Symbol** | **Covid Group Average expression** | **Control Group Average expression** | **Fold Change** | **P-value** |
| --- | --- | --- | --- | --- | --- |
| TC0300014075.hg.1 | **TRIM59** | 1089.92 | 675.59 | **1.62** | 0.0313 |
| TC1400009198.hg.1 | **DDHD1** | 219.79 | 136.24 | **1.62** | 0.0233 |
| TC1500008511.hg.1 | **LRRC28** | 333.14 | 206.50 | **1.62** | 0.0149 |
| TC0300009016.hg.1 | **ATP1B3** | 159.79 | 98.36 | **1.62** | 0.0166 |
| TC0100017212.hg.1 | **SLC30A1** | 85.04 | 52.35 | **1.62** | 0.0145 |
| TC1400007320.hg.1 | **DACT1** | 56.89 | 35.02 | **1.62** | 0.0428 |
| TC0200016452.hg.1 | **REL** | 1105.13 | 680.29 | **1.62** | 0.0022 |
| TC1700008719.hg.1 | **KPNA2** | 315.17 | 194.01 | **1.62** | 0.0033 |
| TC0700010504.hg.1 | **OSBPL3** | 23.26 | 14.42 | **1.61** | 0.0254 |
| TC0600007266.hg.1 | **HIST1H1C** | 143.01 | 88.65 | **1.61** | 0.0067 |
| TC0800011312.hg.1 | **RRM2B** | 328.56 | 203.66 | **1.61** | 0.0194 |
| TC0700008707.hg.1 | **RINT1** | 207.94 | 129.79 | **1.61** | 0.0049 |
| TC2100007663.hg.1 | **BTG3** | 38.59 | 23.92 | **1.61** | 0.0475 |
| TC1700010363.hg.1 | **RP11-466A19.5; MYO1D** | 13.83 | 8.57 | **1.61** | 0.0359 |
| TC2000009053.hg.1 | **TTI1** | 114.56 | 71.01 | **1.61** | 0.0009 |
| TC1100013043.hg.1 | **FAM89B** | 187.40 | 116.16 | **1.61** | 0.0034 |
| TC0200012405.hg.1 | **THADA** | 207.94 | 128.89 | **1.61** | 0.0178 |
| TC1200012624.hg.1 | **TUBA1C** | 3492.39 | 2164.77 | **1.61** | 0.0218 |
| TC0100015743.hg.1 | **ENSA** | 1408.55 | 885.29 | **1.6** | 0.0275 |
| TC0200014512.hg.1 | **ZEB2** | 233.94 | 146.02 | **1.6** | 0.0133 |
| TC1200010655.hg.1 | **LIMA1** | 52.35 | 32.67 | **1.6** | 0.001 |
| TC1100011606.hg.1 | **C2CD3** | 229.13 | 143.01 | **1.6** | 0.0159 |
| TC0800012312.hg.1 | **SGK3** | 88.65 | 55.33 | **1.6** | 0.04 |
| TC1400010592.hg.1 | **NUBPL** | 28.84 | 18.00 | **1.6** | 0.0169 |
| TC1600007030.hg.1 | **NPIPA7; NPIPA8; PKD1P1** | 897.64 | 568.10 | **1.59** | 0.0057 |
| TC0500011418.hg.1 | **MEF2C** | 1332.57 | 843.36 | **1.59** | 0.0337 |
| TC0600010057.hg.1 | **ACAT2** | 43.71 | 27.67 | **1.59** | 0.0217 |
| TC1000009894.hg.1 | **FAM188A** | 103.97 | 65.34 | **1.59** | 0.002 |
| TC0700007285.hg.1 | **CDK13** | 1428.22 | 897.64 | **1.59** | 0.0162 |
| TC0600011145.hg.1 | **HIST1H4H** | 424.61 | 266.87 | **1.59** | 0.02 |
| TC0100008101.hg.1 | **KIF2C** | 24.25 | 15.24 | **1.59** | 0.0482 |
| TC0400008947.hg.1 | **TMEM184C** | 415.87 | 261.38 | **1.59** | 0.0264 |
| TC1700009592.hg.1 | **MED31** | 100.43 | 62.68 | **1.59** | 0.0288 |
| TC1700008499.hg.1 | **BCAS3** | 96.34 | 60.55 | **1.59** | 0.0029 |
| TC0400011748.hg.1 | **CCNA2** | 196.72 | 123.64 | **1.59** | 0.0432 |

| **Probe ID** | **Gene Symbol** | **Covid Group Average expression** | **Control Group Average expression** | **Fold Change** | **P-value** |
| --- | --- | --- | --- | --- | --- |
| TC0600014328.hg.1 | **OSTM1** | 709.18 | 445.72 | **1.59** | 0.0135 |
| TC0200015329.hg.1 | **GTF3C3** | 132.51 | 83.29 | **1.59** | 0.011 |
| TC0300007055.hg.1 | **CTDSPL** | 81.57 | 51.63 | **1.58** | 0.02 |
| TC1000009873.hg.1 | **DCLRE1C** | 385.34 | 243.88 | **1.58** | 0.0009 |
| TC0600007270.hg.1 | **HIST1H2AC** | 2120.22 | 1341.84 | **1.58** | 0.0281 |
| TC1100011794.hg.1 | **PRCP** | 256.00 | 162.02 | **1.58** | 0.0116 |
| TC1500010256.hg.1 | **BTBD1** | 1136.20 | 719.08 | **1.58** | 0.047 |
| TC1500007833.hg.1 | **PML** | 86.82 | 54.95 | **1.58** | 0.038 |
| TC0100015781.hg.1 | **PI4KB** | 263.20 | 166.57 | **1.58** | 0.028 |
| TC1000012577.hg.1 | **LIPA** | 354.59 | 224.41 | **1.58** | 0.0435 |
| TSUnmapped00000205.hg.1 | **SURF4** | 328.56 | 207.94 | **1.58** | 0.0022 |
| TC1600009412.hg.1 | **TXNDC11** | 205.07 | 130.69 | **1.57** | 0.0134 |
| TC0900011454.hg.1 | **ZBTB26** | 34.06 | 21.71 | **1.57** | 0.0108 |
| TC0200015332.hg.1 | **PGAP1** | 31.78 | 20.25 | **1.57** | 0.0163 |
| TC0100007447.hg.1 | **CEP85** | 14.12 | 9.00 | **1.57** | 0.0021 |
| TC1000012130.hg.1 | **FAM53B** | 61.82 | 39.40 | **1.57** | 0.0368 |
| TC1400008913.hg.1 | **SNX6** | 1758.34 | 1136.20 | **1.56** | 0.0304 |
| TC1000007461.hg.1 | **RASSF4** | 105.42 | 67.65 | **1.56** | 0.0444 |
| TC0200009428.hg.1 | **CCNT2** | 3104.19 | 1992.00 | **1.56** | 0.0498 |
| TC1600008712.hg.1 | **IRF8** | 556.41 | 357.05 | **1.56** | 0.0317 |
| TC1800007504.hg.1 | **KIAA1468** | 174.85 | 112.21 | **1.56** | 0.02 |
| TC0400007592.hg.1 | **PAICS** | 235.57 | 151.17 | **1.56** | 0.041 |
| TC1900011682.hg.1 | **RAB8A** | 4039.61 | 2592.27 | **1.56** | 0.0461 |
| TC1700011820.hg.1 | **SRSF2; MIR636** | 367.09 | 235.57 | **1.56** | 0.0328 |
| TC0900010586.hg.1 | **C9orf64** | 71.51 | 45.89 | **1.56** | 0.0451 |
| TC0200011720.hg.1 | **ODC1; SNORA80B** | 40342.14 | 25709.25 | **1.56** | 0.034 |
| TC0500013359.hg.1 | **PPP2CA** | 2665.15 | 1698.45 | **1.56** | 0.0222 |
| TC1200009461.hg.1 | **EP400; SNORA49** | 92.41 | 59.71 | **1.55** | 0.0388 |
| TC0100015254.hg.1 | **LRIF1** | 89.26 | 57.68 | **1.55** | 0.0058 |
| TC0100013323.hg.1 | **STPG1** | 35.26 | 22.78 | **1.55** | 0.0489 |
| TC1000008961.hg.1 | **NHLRC2** | 129.79 | 83.87 | **1.55** | 0.0033 |
| TC1400010624.hg.1 | **PTGR2** | 16.91 | 10.85 | **1.55** | 0.0212 |
| TC1200012240.hg.1 | **RSRC2** | 2320.15 | 1499.22 | **1.55** | 0.0137 |
| TC1300009858.hg.1 | **TUBGCP3** | 55.72 | 35.75 | **1.55** | 0.0187 |
| TC1200011838.hg.1 | **CMKLR1** | 42.81 | 27.47 | **1.55** | 0.0419 |
| TC0X00011226.hg.1 | **F8** | 13.93 | 9.06 | **1.54** | 0.0144 |

| **Probe ID** | **Gene Symbol** | **Covid Group Average expression** | **Control Group Average expression** | **Fold Change** | **P-value** |
| --- | --- | --- | --- | --- | --- |
| TC1100010271.hg.1 | **UEVLD** | 59.30 | 38.32 | **1.54** | 0.0196 |
| TC0300012255.hg.1 | **OSBPL11** | 418.77 | 272.48 | **1.54** | 0.013 |
| TC1600009916.hg.1 | **NPIPB11** | 5330.30 | 3444.31 | **1.54** | 0.0012 |
| TC1000012164.hg.1 | **DHX32** | 74.54 | 48.50 | **1.54** | 0.0157 |
| TC0200007058.hg.1 | **ATRAID** | 680.29 | 439.59 | **1.54** | 0.0254 |
| TC1700007899.hg.1 | **MLX** | 126.24 | 82.14 | **1.54** | 0.0143 |
| TC1400008908.hg.1 | **EAPP** | 421.68 | 274.37 | **1.54** | 0.0209 |
| TC0100009760.hg.1 | **NUDT4P1; NUDT4P2; NUDT4** | 265803.39 | 172950.54 | **1.54** | 0.0443 |
| TC0100009833.hg.1 | **NUDT4; NUDT4P1** | 265803.39 | 172950.54 | **1.54** | 0.0443 |
| TC1100006644.hg.1 | **RRM1** | 257.78 | 167.73 | **1.54** | 0.0375 |
| TC0100016000.hg.1 | **MEF2D** | 114.56 | 74.03 | **1.54** | 0.0215 |
| TC0100017241.hg.1 | **TMEM206** | 28.64 | 18.77 | **1.53** | 0.0098 |
| TC1100008435.hg.1 | **RNF169** | 229.13 | 150.12 | **1.53** | 0.0164 |
| TC1100013110.hg.1 | **STT3A** | 1795.29 | 1176.27 | **1.53** | 0.0278 |
| TC1900009867.hg.1 | **AKAP8L** | 1351.18 | 885.29 | **1.53** | 0.0455 |
| TC0200011965.hg.1 | **ATAD2B** | 202.25 | 132.51 | **1.53** | 0.0308 |
| TC1300007969.hg.1 | **ABHD13** | 515.56 | 337.79 | **1.53** | 0.0493 |
| TC1900009128.hg.1 | **TMEM259** | 191.34 | 125.37 | **1.53** | 0.0499 |
| TC0600011224.hg.1 | **HIST1H2BL** | 26.72 | 17.39 | **1.53** | 0.0226 |
| TC1300008468.hg.1 | **SLC46A3** | 106.15 | 69.07 | **1.53** | 0.0304 |
| TC0100015696.hg.1 | **HIST2H2BF** | 1710.26 | 1128.35 | **1.52** | 0.0198 |
| TC0400008213.hg.1 | **RAP1GDS1** | 23.75 | 15.67 | **1.52** | 0.0223 |
| TC0900011013.hg.1 | **ERP44** | 719.08 | 474.41 | **1.52** | 0.0077 |
| TC0500010731.hg.1 | **ARL15** | 203.66 | 134.36 | **1.52** | 0.0275 |
| TC0900011962.hg.1 | **NACC2** | 29.86 | 19.56 | **1.52** | 0.0234 |
| TC1800008779.hg.1 | **NARS** | 680.29 | 445.72 | **1.52** | 0.0471 |
| TC0300008018.hg.1 | **C3orf38** | 150.12 | 99.04 | **1.52** | 0.0111 |
| TC1100008010.hg.1 | **CDC42EP2** | 152.22 | 100.43 | **1.52** | 0.0092 |
| TC0300011383.hg.1 | **PSMD6** | 445.72 | 292.04 | **1.52** | 0.0345 |
| TC0200015463.hg.1 | **WDR12** | 61.82 | 40.79 | **1.52** | 0.045 |
| TC0100007540.hg.1 | **THEMIS2** | 64.00 | 42.22 | **1.52** | 0.0427 |
| TC0900007520.hg.1 | **SMC5** | 150.12 | 98.36 | **1.52** | 0.0418 |
| TC1300008289.hg.1 | **ZDHHC20** | 1016.93 | 666.29 | **1.52** | 0.0361 |
| TC0X00010130.hg.1 | **MAGT1** | 968.76 | 643.59 | **1.51** | 0.0352 |
| TC0600014084.hg.1 | **HIST1H4I** | 98.36 | 65.34 | **1.51** | 0.0437 |
| TC0X00009124.hg.1 | **PIGA** | 95.67 | 63.56 | **1.51** | 0.0086 |
| TC1900009592.hg.1 | **ZNF562** | 165.42 | 109.14 | **1.51** | 0.0173 |

| **Probe ID** | **Gene Symbol** | **Covid Group Average expression** | **Control Group Average expression** | **Fold Change** | **P-value** |
| --- | --- | --- | --- | --- | --- |
| TC1700009368.hg.1 | **YWHAE** | 1234.75 | 820.30 | **1.51** | 0.0195 |
| TC0600014126.hg.1 | **GLTSCR1L** | 153.28 | 101.13 | **1.51** | 0.0222 |
| TC0100016639.hg.1 | **SHCBP1L** | 10.34 | 6.87 | **1.51** | 0.0237 |
| TC0400011721.hg.1 | **MAD2L1** | 32.22 | 21.41 | **1.51** | 0.0025 |
| TC0500012147.hg.1 | **HNRNPA0** | 206.50 | 137.19 | **1.51** | 0.0089 |
| TC1900010574.hg.1 | **ZNF571** | 71.01 | 46.85 | **1.51** | 0.0393 |
| TC0200008262.hg.1 | **RNF181** | 40.79 | 27.10 | **1.51** | 0.0281 |
| TC0300009168.hg.1 | **SELT; SELT.1** | 13216.02 | 8719.32 | **1.51** | 0.0258 |
| TC0100018257.hg.1 | **GNAI3** | 1663.49 | 1105.13 | **1.51** | 0.0278 |
| TC1100008480.hg.1 | **UVRAG** | 252.48 | 167.73 | **1.51** | 0.0435 |
| TC0600007616.hg.1 | **HSPA1B; HSPA1A** | 191.34 | 127.12 | **1.51** | 0.0378 |
| TC1200011400.hg.1 | **CCDC59** | 455.09 | 302.33 | **1.51** | 0.0445 |
| TC0900011519.hg.1 | **PPP6C** | 2256.70 | 1488.87 | **1.51** | 0.047 |
| TSUnmapped00000258.hg.1 | **SURF4** | 147.03 | 97.01 | **1.51** | 0.0113 |
| TC0X00010356.hg.1 | **BTK** | 261.38 | 173.65 | **1.5** | 0.0357 |
| TC1100008067.hg.1 | **RAB1B** | 6936.54 | 4608.24 | **1.5** | 0.0471 |
| TC0300008542.hg.1 | **CD86** | 92.41 | 61.39 | **1.5** | 0.0479 |
| TC0400010745.hg.1 | **CLOCK** | 31.34 | 20.82 | **1.5** | 0.0168 |
| TC1400008684.hg.1 | **PRMT5** | 60.55 | 40.22 | **1.5** | 0.0113 |
| TC1700009260.hg.1 | **FOXK2** | 699.41 | 464.65 | **1.5** | 0.0139 |
| TC0800007529.hg.1 | **SPIDR** | 117.78 | 78.25 | **1.5** | 0.0137 |
