# Supplementary material for: COVID-19 Infection During Pregnancy Induces Differential Gene Expression in Human Cord Blood Cells From Term Neonates
Source: Front Pediatr. 2022 Apr 25;10:834771. doi: 10.3389/fped.2022.834771 (PMC9084610; doi:10.3389/fped.2022.834771)
Supplement: Supplementary Table 2 — Differentially Downregulated ProbeID or Genes in COVID Group. [file Table_2.DOCX]

**Supplementary Table-2: Differentially Downregulated ProbeID or Genes in COVID Group:**

| **Probe ID** | **Gene Symbol** | **Covid Group Average expression** | **Control Group Average expression** | **Fold Change** | **P-value** |
| --- | --- | --- | --- | --- | --- |
| TC1600011312.hg.1 | **HBZ** | 4640.29 | 56266.94 | -12.09 | 0.0148 |
| TC0X00007704.hg.1 | **COX7B** | 699.41 | 2062.24 | -2.94 | 0.0448 |
| TC0200008351.hg.1 | **RPIA** | 4039.61 | 11585.24 | -2.86 | 0.0484 |
| TC1400007430.hg.1 | **SYNE2** | 39.40 | 105.42 | -2.68 | 0.0222 |
| TC0100018307.hg.1 | **ACKR1** | 14.93 | 37.53 | -2.51 | 0.0411 |
| TC0700009680.hg.1 | **TMEM176A** | 25.11 | 55.33 | -2.19 | 0.0089 |
| TC0800006692.hg.1 | **MSRA** | 39.67 | 85.63 | -2.16 | 0.0061 |
| TC0900007457.hg.1 | **CNTNAP3P2; CNTNAP3** | 59.30 | 127.12 | -2.15 | 0.0279 |
| TC0X00011308.hg.1 | **RPL36A** | 3590.58 | 7486.11 | -2.09 | 0.0279 |
| TC1000008159.hg.1 | **RPS24** | 82952.62 | 172950.54 | -2.08 | 0.0253 |
| TC1300007925.hg.1 | **DAOA** | 6.96 | 13.55 | -1.95 | 0.0072 |
| TC1400007227.hg.1 | **LGALS3** | 4039.61 | 7858.29 | -1.95 | 0.0194 |
| TC0200016424.hg.1 | **LBH** | 1052.79 | 2048.00 | -1.94 | 0.0407 |
| TC1200012799.hg.1 | **SARNP** | 51.63 | 98.36 | -1.91 | 0.0112 |
| TC0500011520.hg.1 | **ERAP1** | 12.82 | 24.59 | -1.91 | 0.0018 |
| TC0200016501.hg.1 | **IL1RL1** | 21.71 | 41.07 | -1.89 | 0.0046 |
| TC1100011545.hg.1 | **ANAPC15** | 82.71 | 154.34 | -1.87 | 0.007 |
| TC0100007326.hg.1 | **RPL11** | 31433.17 | 58251.19 | -1.86 | 0.0375 |
| TC0900011576.hg.1 | **RPL12** | 23.75 | 43.41 | -1.83 | 0.0051 |
| TC0X00008259.hg.1 | **NDUFA1** | 544.96 | 996.00 | -1.83 | 0.0155 |
| TC0500011220.hg.1 | **AP3B1** | 8.63 | 15.78 | -1.82 | 0.0093 |
| TC0100012454.hg.1 | **SDF4** | 76.64 | 139.10 | -1.82 | 0.0243 |
| TC0X00006775.hg.1 | **PHEX** | 10.27 | 18.51 | -1.8 | 0.011 |
| TC0800009494.hg.1 | **DEFB109P1B** | 11.31 | 20.39 | -1.8 | 0.0143 |
| TC1400009682.hg.1 | **ENTPD5** | 59.71 | 107.63 | -1.8 | 0.0165 |
| TC0500010780.hg.1 | **SLC38A9** | 27.86 | 49.87 | -1.79 | 0.0017 |
| TC0X00011310.hg.1 | **RPL36A-HNRNPH2** | 1758.34 | 3147.52 | -1.79 | 0.031 |
| TC0800009919.hg.1 | **GNRH1** | 74.03 | 131.60 | -1.77 | 0.0025 |
| TC0800007592.hg.1 | **SNTG1** | 8.06 | 14.22 | -1.76 | 0.0013 |
| TC0800009735.hg.1 | **MTUS1** | 194.01 | 340.14 | -1.75 | 0.0305 |
| TC1900009637.hg.1 | **CDKN2D** | 526.39 | 916.51 | -1.74 | 0.0371 |
| TC1900011236.hg.1 | **KLK13** | 22.16 | 38.59 | -1.74 | 0.0012 |
| TC0700011905.hg.1 | **BAIAP2L1** | 10.27 | 17.75 | -1.73 | 0.003 |
| TC1800008387.hg.1 | **DSC1** | 48.84 | 83.87 | -1.73 | 0.0472 |
| TC0500013298.hg.1 | **C1QTNF3** | 8.46 | 14.32 | -1.7 | 0.0216 |
| TC0100015993.hg.1 | **VHLL** | 7.41 | 12.47 | -1.68 | 0.0018 |
| **Probe ID** | **Gene Symbol** | **Covid Group Average expression** | **Control Group Average expression** | **Fold Change** | **P-value** |
| TC1100011052.hg.1 | **FADS3** | 47.84 | 79.89 | -1.68 | 0.0268 |
| TC2100007166.hg.1 | **SH3BGR** | 25.81 | 43.11 | -1.67 | 0.0036 |
| TC0300012269.hg.1 | **ALDH1L1** | 24.08 | 40.22 | -1.67 | 0.0129 |
| TC0X00011132.hg.1 | **CSAG3** | 19.16 | 32.00 | -1.67 | 0.0027 |
| TC0700012824.hg.1 | **MRPS33** | 46.53 | 77.17 | -1.66 | 0.0297 |
| TC1900006890.hg.1 | **RPS28** | 5404.70 | 8964.45 | -1.66 | 0.0479 |
| TC1100008321.hg.1 | **DEFB108B** | 15.45 | 25.63 | -1.65 | 0.0026 |
| TC0300007448.hg.1 | **IQCF3** | 7.36 | 12.13 | -1.65 | 0.0299 |
| TC0X00010204.hg.1 | **SATL1** | 8.63 | 14.22 | -1.65 | 0.0021 |
| TC0500010753.hg.1 | **CDC20B** | 12.64 | 20.82 | -1.65 | 0.0346 |
| TC1600011406.hg.1 | **CKLF-CMTM1** | 31.34 | 51.63 | -1.65 | 0.0119 |
| TC1100009997.hg.1 | **ZNF214** | 21.11 | 34.78 | -1.64 | 0.0037 |
| TC0300008098.hg.1 | **OR5H14** | 6.68 | 10.93 | -1.64 | 0.0487 |
| TC0800007321.hg.1 | **ASH2L** | 879.17 | 1438.15 | -1.64 | 0.0428 |
| TC2000007479.hg.1 | **PI3** | 20.53 | 33.82 | -1.64 | 0.0465 |
| TC1700010068.hg.1 | **CCDC144NL** | 58.08 | 95.01 | -1.64 | 0.004 |
| TC0200016649.hg.1 | **CDC42EP3** | 11.55 | 18.77 | -1.63 | 0.0128 |
| TC1600011306.hg.1 | **PRDM7** | 20.97 | 34.06 | -1.63 | 0.0087 |
| TC0300013807.hg.1 | **XYLB** | 30.06 | 48.84 | -1.62 | 0.0057 |
| TC1900011755.hg.1 | **ZNF226** | 53.82 | 87.43 | -1.62 | 0.0283 |
| TC0X00006935.hg.1 | **MAGEB16** | 9.45 | 15.35 | -1.62 | 0.0002 |
| TC1300007972.hg.1 | **TNFSF13B** | 21.41 | 34.78 | -1.62 | 0.0024 |
| TC0200014800.hg.1 | **SLC38A11** | 6.15 | 9.99 | -1.62 | 0.0033 |
| TC0300012073.hg.1 | **LSAMP** | 11.31 | 18.25 | -1.62 | 0.0066 |
| TC0500013215.hg.1 | **LVRN** | 33.13 | 53.45 | -1.62 | 0.023 |
| TC0200013351.hg.1 | **RGPD1; RGPD2** | 52.71 | 85.04 | -1.62 | 0.0414 |
| TC0200011382.hg.1 | **SNED1** | 101.13 | 163.14 | -1.62 | 0.0211 |
| TC2100006878.hg.1 | **KRTAP6-3** | 32.67 | 52.71 | -1.61 | 0.0459 |
| TC0X00007325.hg.1 | **RIBC1** | 45.57 | 73.52 | -1.61 | 0.0028 |
| TC0200015204.hg.1 | **COL5A2** | 15.67 | 25.28 | -1.61 | 0.0002 |
| TC1100009912.hg.1 | **OR52A1** | 9.13 | 14.72 | -1.6 | 0.007 |
| TC1100008612.hg.1 | **RPS28** | 13777.25 | 22073.07 | -1.6 | 0.0391 |
| TC0400008624.hg.1 | **SPATA5** | 22.78 | 36.50 | -1.6 | 0.0235 |
| TC1700006808.hg.1 | **RANGRF** | 32.90 | 52.71 | -1.6 | 0.001 |
| TC0X00010172.hg.1 | **HMGN5** | 11.71 | 18.77 | -1.6 | 0.0181 |
| TC1100011295.hg.1 | **CCDC87** | 20.68 | 33.13 | -1.6 | 0.0255 |
| TC0800012185.hg.1 | **OPLAH** | 47.50 | 76.11 | -1.6 | 0.0029 |
| TC0300012319.hg.1 | **goyborbu; MCM2** | 8.69 | 13.93 | -1.6 | 0.0037 |
| TC0500010282.hg.1 | **CDH12** | 18.64 | 29.65 | -1.59 | 0.0026 |
| **Probe ID** | **Gene Symbol** | **Covid Group Average expression** | **Control Group Average expression** | **Fold Change** | **P-value** |
| TC0100015060.hg.1 | **LRRC39** | 40.79 | 64.89 | -1.59 | 0.0152 |
| TC1600006788.hg.1 | **RBFOX1** | 133.44 | 210.84 | -1.59 | 0.0198 |
| TC0300011223.hg.1 | **SELK** | 948.83 | 1499.22 | -1.59 | 0.027 |
| TC1400006446.hg.1 | **OR11H12** | 7.06 | 11.16 | -1.58 | 0.0489 |
| TC0900009003.hg.1 | **POMT1** | 64.45 | 101.83 | -1.58 | 0.011 |
| TC1100006746.hg.1 | **OR10A2** | 8.57 | 13.55 | -1.58 | 0.0036 |
| TC0200010438.hg.1 | **NDUFB3** | 265.03 | 418.77 | -1.58 | 0.0392 |
| TC2100007886.hg.1 | **KRTAP27-1** | 27.47 | 43.41 | -1.58 | 0.0076 |
| TC1000012552.hg.1 | **FAM25G; FAM25C; FAM25BP** | 44.94 | 70.52 | -1.57 | 0.0017 |
| TC1500009505.hg.1 | **PRTG** | 12.04 | 18.90 | -1.57 | 0.0059 |
| TC1400007733.hg.1 | **LRRC74A** | 21.11 | 33.13 | -1.57 | 0.0074 |
| TC0800009468.hg.1 | **DEFB106B; DEFB106A** | 11.16 | 17.51 | -1.57 | 0.0249 |
| TC0400008359.hg.1 | **RPL34** | 24661.96 | 38698.77 | -1.57 | 0.0378 |
| TC2100007506.hg.1 | **C21orf140** | 16.56 | 25.81 | -1.56 | 0.041 |
| TC2100008025.hg.1 | **C21orf140** | 16.56 | 25.81 | -1.56 | 0.041 |
| TC0800008658.hg.1 | **COLEC10** | 15.24 | 23.75 | -1.56 | 0.0049 |
| TC1000009922.hg.1 | **VIM** | 51.63 | 80.45 | -1.56 | 0.0148 |
| TC1100009898.hg.1 | **OR51H1** | 10.93 | 17.03 | -1.56 | 0.0086 |
| TC1700011574.hg.1 | **ABCA8** | 14.22 | 22.01 | -1.56 | 0.0272 |
| TC0800009212.hg.1 | **MAPK15** | 22.47 | 34.78 | -1.55 | 0.0113 |
| TC0300013602.hg.1 | **ATP13A3** | 14.52 | 22.47 | -1.55 | 0.043 |
| TC0600011294.hg.1 | **OR2B3** | 7.52 | 11.63 | -1.55 | 0.0048 |
| TC1100007951.hg.1 | **CCDC88B** | 31.56 | 48.84 | -1.55 | 0.008 |
| TC0100018176.hg.1 | **FHAD1** | 15.56 | 24.08 | -1.55 | 0.0075 |
| TC1100012353.hg.1 | **ZW10** | 25.99 | 39.95 | -1.55 | 0.046 |
| TC0X00009793.hg.1 | **PFKFB1** | 13.36 | 20.53 | -1.54 | 0.0021 |
| TC1700010709.hg.1 | **KAT2A** | 31.12 | 47.84 | -1.54 | 0.0253 |
| TC0600013588.hg.1 | **SYNE1** | 91.77 | 142.02 | -1.54 | 0.046 |
| TC0300008268.hg.1 | **CCDC54** | 7.62 | 11.79 | -1.54 | 0.0416 |
| TC1800007448.hg.1 | **GRP** | 34.30 | 52.71 | -1.54 | 0.0069 |
| TC1600009664.hg.1 | **ACSM1** | 8.11 | 12.55 | -1.54 | 0.0204 |
| TC1600008766.hg.1 | **FBXO31** | 8.94 | 13.83 | -1.54 | 0.0014 |
| TC0X00006489.hg.1 | **ARSF** | 12.82 | 19.70 | -1.54 | 0.0118 |
| TC1900011785.hg.1 | **NR1H2** | 9.92 | 15.35 | -1.54 | 0.003 |
| TC0300010668.hg.1 | **SUSD5** | 12.82 | 19.56 | -1.53 | 0.0165 |
| TC1100007780.hg.1 | **MS4A10** | 9.13 | 13.93 | -1.53 | 0.0083 |
| TC0700007475.hg.1 | **C7orf57** | 10.06 | 15.45 | -1.53 | 0.0002 |
| TC1500007805.hg.1 | **BBS4** | 13.74 | 21.11 | -1.53 | 0.0363 |
| **Probe ID** | **Gene Symbol** | **Covid Group Average expression** | **Control Group Average expression** | **Fold Change** | **P-value** |
| TC0600008505.hg.1 | **RIMS1** | 12.47 | 19.16 | -1.53 | 0.0296 |
| TC0400012950.hg.1 | **RP11-499E18.1; SLC39A8** | 8.22 | 12.55 | -1.53 | 0.0015 |
| TC0800010034.hg.1 | **GSR** | 776.05 | 1184.45 | -1.53 | 0.0343 |
| TC0600012647.hg.1 | **FBXL4** | 867.07 | 1323.37 | -1.52 | 0.0118 |
| TC1500010745.hg.1 | **POLR2M** | 8.75 | 13.36 | -1.52 | 0.0104 |
| TC2200008576.hg.1 | **RBFOX2** | 9.58 | 14.52 | -1.52 | 0.0261 |
| TC0300009189.hg.1 | **AADACL2** | 7.36 | 11.16 | -1.52 | 0.012 |
| TC0600011957.hg.1 | **ADGRF1** | 12.64 | 19.16 | -1.52 | 0.0462 |
| TC0300007335.hg.1 | **TMA7** | 544.96 | 826.00 | -1.52 | 0.0295 |
| TC0300008140.hg.1 | **TMEM30C** | 9.32 | 14.12 | -1.52 | 0.0231 |
| TC0200015971.hg.1 | **SLC16A14** | 37.27 | 56.49 | -1.51 | 0.0093 |
| TC0200015603.hg.1 | **ACADL** | 16.34 | 24.59 | -1.51 | 0.0186 |
| TC1300009980.hg.1 | **LMO7** | 17.63 | 26.72 | -1.51 | 0.0292 |
| TC0500009801.hg.1 | **EXOC3-AS1** | 31.34 | 47.50 | -1.51 | 0.0044 |
| TC1300006543.hg.1 | **FGF9** | 47.50 | 72.00 | -1.51 | 0.0372 |
| TC0700012798.hg.1 | **KDM7A** | 2418.67 | 3640.70 | -1.51 | 0.0318 |
| TC0700010671.hg.1 | **PDE1C** | 13.00 | 19.56 | -1.51 | 0.038 |
| TC0400012305.hg.1 | **NPY1R** | 7.57 | 11.47 | -1.51 | 0.0477 |
| TC0100012938.hg.1 | **HNRNPCL2** | 8.40 | 12.64 | -1.51 | 0.0046 |
| TC0900006442.hg.1 | **KANK1** | 7.67 | 11.55 | -1.51 | 0.0073 |
| TC1400010049.hg.1 | **SERPINA12** | 5.78 | 8.69 | -1.5 | 0.0432 |
| TC0500009478.hg.1 | **RPL26L1** | 35.26 | 52.71 | -1.5 | 0.0251 |
| TC1800007682.hg.1 | **CNDP1** | 7.89 | 11.79 | -1.5 | 0.0016 |
